# Stage-specific biomimetic nanoparticles reprogram osteoblast-adipocyte equilibrium for targeted osteoporosis therapy

**DOI:** 10.1016/j.bioactmat.2026.05.004

**Published:** 2026-05-13

**Authors:** Kehan Cai, Zhe Fan, Jun Tan, Wei Cai, Minghang Zhang, Haojie Tang, Jianzhong Xu, Han Lu, Wei He, Qian Xu, Yazhou Chen, Tao Chen

**Affiliations:** a Department of Orthopedic Surgery, The First Affiliated Hospital of Zhengzhou University, Zhengzhou, 450052, China; b Henan Institute of Advanced Technology, Zhengzhou University, Zhengzhou, 450001, China; c Department of Mini-invasive Spinal Surgery, The Third People's Hospital of Henan Province, Zhengzhou, 450000, China; d Department of Orthopedics, The Third People's Hospital of Henan Province, Zhengzhou, 450052, China; e Department of Oral and Maxillofacial Surgery, The First Affiliated Hospital of Zhengzhou University, Zhengzhou University, Zhengzhou, 450052, China; f College of Life Science, Nanyang Normal University, Nanyang, 473061, China

**Keywords:** Stepwise, Osteogenic differentiation, Biomimetic nanoparticle, Osteoporosis, Bone formation

## Abstract

Cell membrane-camouflaged nanoparticles have emerged as powerful tools for targeted drug delivery; however, current strategies typically utilize membranes from static cell states, overlooking the dynamic functional evolution that occurs during lineage commitment. Here, we established a stepwise osteogenic differentiation model for BMSCs and isolated cell membranes from distinct stages of this process to construct a series of cell membrane–camouflaged mesoporous silica nanoparticles (CM-MSNs). Proteomic profiling revealed stage-dependent remodeling of membrane protein composition, with early osteogenic (EO) stage membranes uniquely enriched in phosphatases and cadherins. Functional evaluations showed stage-dependent activity among CM-MSNs, and EO membrane-camouflaged nanoparticles (EO-MSNs) exhibited the strongest capacity to promote calcium deposition and enhance BMSC osteogenesis *in vitro* via the Wnt/β-catenin signaling pathway. In a rat model of osteoporosis, EO-MSN exhibited prolonged circulation time, precise bone-specific accumulation, and potent anti-osteoporotic efficacy. Collectively, our findings suggest that utilizing stage-specific cell membranes offers a novel strategy to remodel the osteoporotic microenvironment by modulating the osteoblast-adipocyte equilibrium.

## Highlights

• Stage-specific membranes reveal dynamic evolution of proteome during osteogenesis.
• EO-MSNs activate Wnt/β-catenin pathway to enhance osteogenesis *in vitro.*
• EO-MSNs achieve targeted bone accumulation and extended circulation *in vivo.*
• Stage-specific membranes reprogram osteoblast-adipocyte balance in osteoporosis.

## Graphical abstract

**Figure:**
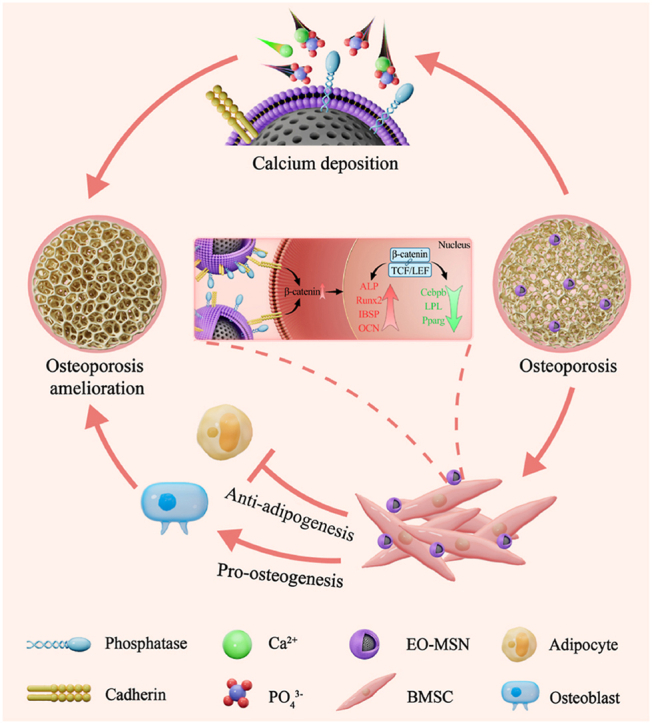

## 1 Introduction

Osteoporosis (OP) is a prevalent chronic skeletal disorder caused by an imbalance between bone formation and resorption [1]. This condition leads to a pathological bone microenvironment characterized by oxidative stress, impaired bone and immune cell homeostasis, disrupted signaling pathways, and altered inorganic composition [[2], [3], [4]]. Extensive studies have reported that Wnt/β-catenin signaling is frequently dysregulated or suppressed in the osteoporotic microenvironment, leading to impaired osteogenic commitment of bone marrow mesenchymal stem cells (BMSCs) and a shift toward adipogenic differentiation [5,6]. This phenotypic shift reduces bone formation and accelerates the progression of OP. Current clinical treatments for OP mainly include anti-resorptive and anabolic agents, such as bisphosphonates, estrogen, and teriparatide [[7], [8], [9]]. However, these therapeutic approaches are often associated with considerable side effects and do not adequately correct the dysregulated differentiation of BMSCs within the pathological bone microenvironment. Moreover, these agents exhibit limited accumulation within the bone microenvironment, which further aggravates the conflict between therapeutic efficacy and side effects [10,11]. Therefore, there is an urgent need for more targeted and effective treatment options to restore this fundamental imbalance and improve clinical outcomes.

Previous studies have demonstrated the therapeutic potential of both allogeneic and autologous BMSC transplantation for osteoporosis treatment [[12], [13], [14], [15]]. However, the potential risks of cellular mutations, uncontrolled cell proliferation, and adverse immune responses following transplantation remain major obstacles to the widespread application of cell-based therapies [16,17]. To overcome these limitations, recent research has concentrated on the use of cell membranes. These membranes exhibit non-proliferative characteristics and reduced immunogenicity, thereby significantly improving their safety profile *in vivo* [18,19]. Furthermore, as these membranes retain surface proteins from the original cells, they can execute specific biological functions contingent upon their cellular origin [20,21]. For instance, Hatano et al. reported that nanofragments derived from the plasma membrane of chondrocytes significantly promoted rapid bone repair within two weeks [22]. Similarly, Yang et al. developed an innovative nanoplatform based on membranes from chimeric antigen receptor (CAR)-engineered MSCs, combining the natural tumor-homing ability of MSCs with CAR-mediated specificity to achieve precise and effective tumor targeting [23]. In addition to cell source, the physiological state of donor cells can also significantly influence membrane composition and function. Chen et al. reported that tumor cells treated with liposomal doxorubicin exhibited increased expression of membrane-associated immune molecules, which enhanced the activation and maturation of immune cells and demonstrated superior efficacy in preventing relapse and metastasis in cancer models [24].

Membrane-based therapies rely heavily on the origin and physiological state of the source cells. BMSCs, which are pivotal for bone regeneration, undergo distinct temporal stages during osteogenesis [[25], [26], [27], [28]]. Although the overall cellular changes throughout this stepwise process are well recognized, the specific molecular evolution of cellular components remains complex and largely unexplored. Our previous studies showed that the composition and function of the extracellular matrix (ECM) undergo dynamic remodeling across different stages of osteogenic differentiation, which in turn exerts stage-dependent regulatory effects on BMSC fate [29]. However, despite these insights into the microenvironmental regulation, whether the plasma membrane itself undergoes analogous stage-specific remodeling during differentiation remains unexplored. Bridging this knowledge gap may reveal new strategies for enhancing the functional performance of BMSC membrane-derived therapeutic systems.

**Scheme 1:**
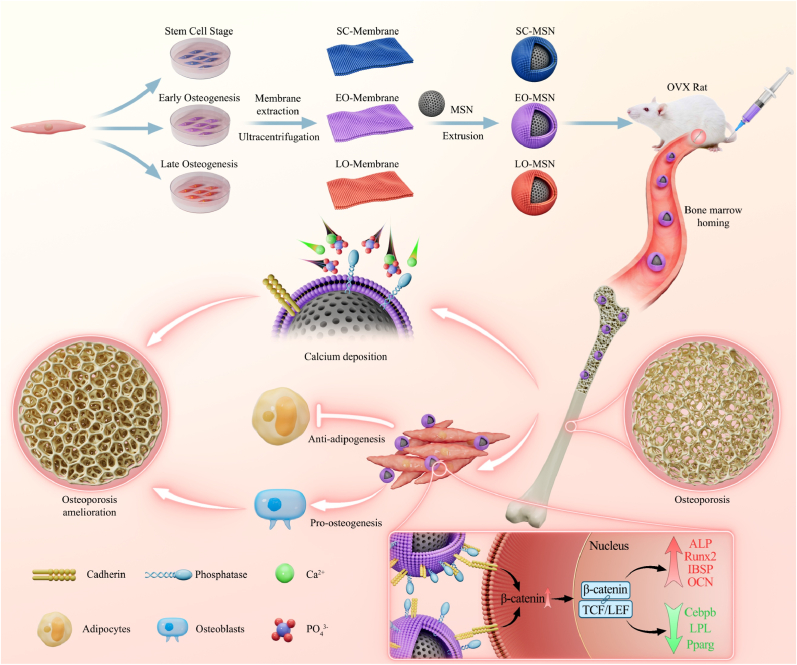
Schematic illustration of the fabrication and therapeutic mechanism of stage-specific membrane-camouflaged MSNs.

Herein, we established a stepwise osteogenic differentiation model for BMSCs and, for the first time, delineated the dynamic evolution of their membrane protein profile throughout this process. Building on these findings, we fabricated differentiation stage–specific membrane-camouflaged mesoporous silica nanoparticles (CM-MSNs). Due to the excellent stability and biocompatibility, with negligible effects on the osteogenic differentiation of stem cells, MSNs were selected as the ideal nanoparticle core to minimize confounding factors in our study [30,31]. *In vitro* experiments were conducted to investigate the regulatory effects of CM-MSNs on the osteogenic differentiation of BMSCs. Upon systemic administration, CM-MSNs exhibited enhanced accumulation in bone tissue, owing to the intrinsic homing capabilities of BMSC membranes. Mechanistically, membrane-anchored phosphatases functioned as bioactive catalytic centers to enhance calcium deposition, thereby mitigating mineral loss and promoting bone formation. Concurrently, cadherins enriched on the nanoparticle surface facilitated direct interactions with resident BMSCs, activating the Wnt/β-catenin signaling pathway to promote osteogenic differentiation while suppressing adipogenesis (Scheme 1).

## 2 Materials and methods

### 2.1 Materials

Rat bone marrow mesenchymal stem cells (BMSCs) were obtained from Pricella (Wuhan, China). α-MEM and fetal bovine serum were purchased from Thermo Fisher Scientific (MA, USA). PBS and penicillin-streptomycin mixed solution were obtained from Servicebio (Wuhan, China). Antibodies against the target proteins are listed in Table S1, and primer sequences are listed in Table S2. All other required chemicals were obtained from commercial suppliers and complied with the standards of analytical grade.

### 2.2 Animal experiments

Six-week-old female Sprague-Dawley (SD) rats were purchased from the Animal Center of Zhengzhou University. All experimental procedures were conducted following the guidelines of the Institutional Animal Care and Use Committee of Zhengzhou University (approval number: ZZU-LAC20240531 [13]).

### 2.3 Cell culture and differentiation

All subsequent *in vitro* experiments were conducted using third-passage rat bone marrow mesenchymal stem cells (BMSCs). Undifferentiated BMSCs were cultured in α-MEM. Osteogenic differentiation was induced by culturing the cells in osteogenic differentiation medium for 7 days to obtain cells at the early osteogenic stage and for 21 days to obtain cells at the late osteogenic stage. The osteogenic differentiation medium consisted of α-MEM, 1% penicillin-streptomycin mixed solution, 10% fetal bovine serum, 10 nM dexamethasone (GLPBIO, GC40775), 10 mM β-glycerophosphate (GLPBIO, GC11806), and 1.5 mM calcium chloride (Aladdin, C431199). Adipogenic differentiation was induced by culturing BMSCs in adipogenic differentiation medium for 14 days. The adipogenic differentiation medium was composed of α-MEM, 1% penicillin-streptomycin mixed solution, 10% fetal bovine serum, 0.1 mM indomethacin (GLPBIO, GC17556), 0.05 mM IBMX (GLPBIO, GC11730), 1 mg/mL insulin (Pricella, PB180432), and 1 μM dexamethasone.

### 2.4 Alkaline phosphatase staining and quantitative analysis

Cells were washed with PBS and fixed with 4% paraformaldehyde (LEAGENE, DF0135) for 15 min. After removal of paraformaldehyde, the alkaline phosphatase (ALP) staining working solution (Beyotime, C3206) was added, and the cells were incubated at room temperature for 1 h. Microscopic examination was subsequently performed.

Quantitative analysis of ALP was performed using the Alkaline Phosphatase Assay Kit (Beyotime, P0321S). The absorbance at 405 nm was converted to p-nitrophenol (pNP) concentration using a standard curve. The enzyme activity was calculated after incubation at 37 °C for 20 min and subsequently normalized to the total protein content determined by a BCA assay. The results were expressed as U/mg protein:

$$\text{ALP}\;\left(U/\text{mg}\;\text{protein}\right)=\frac{\left(OD‐b\right)}{k}\times \frac{V_{total}}{t\cdot V_{sample}\cdot Protein}$$

where *OD* is the absorbance at 405 nm, *b* and *k* are the intercept and slope of the standard curve, respectively, *V*_*total*_ is the total reaction volume (mL), *V*_*sample*_ is the sample volume (mL), *t* is the incubation time (min), and *Protein* (mg/mL) is the protein concentration of the sample determined by the BCA assay.

### 2.5 Alizarin Red S staining and quantitative analysis

Cells were washed with PBS and fixed with 4% paraformaldehyde for 15 min. After the paraformaldehyde was thoroughly washed away, the Alizarin Red S (ARS) staining solution (Beyotime, C0138) was added, and the cells were incubated at room temperature for 30 min. The stained cells were then observed under a microscope. After ARS staining, 10% cetylpyridinium chloride solution (Aladdin, C129534) was used to elute the bound dye. The optical density (OD) of the eluent was subsequently measured at 560 nm to quantify mineralization.

### 2.6 Isolation of cell membranes

To prepare the starting buffer (SB), 4.1 g of mannitol, 2.6 g of sucrose, and 3 mL of Tris-HCl were added to 80 mL of ultrapure water. After complete dissolution, the solution was incubated at 4 °C for 30 min. The pH was then adjusted to 7.4, and the volume was brought to 100 mL. To formulate the isolation buffer (IB), 0.5 g of bovine serum albumin (BSA), 500 μl of EGTA (100 mM), and 100 μl of PMSF (100 mM) were added to SB.

To isolate the membranes of stem cells, the cells were washed with PBS and scraped on ice using a cell scraper. The cells were then centrifuged at 1000 rpm, 4 °C for 5 min. After centrifugation, the cell pellet was resuspended in 4 mL IB in a 10 mL tube and ultrasonicated at 30 W for 5 min in an ice-water bath. The sample was subsequently centrifuged at 3000 × g, 4 °C for 5 min, and the supernatant was collected. The collected supernatant was centrifuged again at 10000 × g, 4 °C for 10 min, and the resulting supernatant was collected again. Subsequently, the obtained supernatant was ultracentrifuged at 100000 × g, 4 °C for 2 h. The resulting precipitate was resuspended in PBS, and the membrane content was quantified using the BCA kit (Beyotime, P0009). The membranes were stored at −80 °C until further use. All procedures were conducted using sterile reagents and consumables under aseptic conditions to prevent contamination.

### 2.7 Preparation of MSNs and membrane-camouflaged MSNs

The synthesis of mesoporous silica nanoparticles (MSNs) was initiated by combining 2 mL of ammonium hydroxide (Aladdin, A112079) with 0.728 g of cetyltrimethylammonium bromide (CTAB, Aladdin, H108983) in 200 mL of ultrapure water. The mixture was stirred at 40 °C for 1 h. Subsequently, 4.167 g of tetraethyl orthosilicate (TEOS, Aladdin, T110595) was dissolved in 100 mL ethanol (Macklin, E809056) and added to the above aqueous solution. The resulting mixture was stirred at 40 °C for 5 h. The product was then collected by centrifugation at 12,000 rpm. The obtained MSNs were calcined in a muffle furnace at 550 °C for 4 h to remove CTAB. Finally, the MSNs were sterilized under ultraviolet (UV) irradiation for subsequent *in vitro* cellular experiments. The cell membrane and MSNs were mixed at a weight ratio of 5:2 and extruded through a 400 nm liposome extruder (Genizer, HandExtruder) for 20 cycles to produce cell membrane-camouflaged MSNs (CM-MSNs).

### 2.8 Characterization of CM-MSNs

The size and zeta potential of CM-MSNs were measured using a nanoparticle size and zeta potential analyzer (OMEC, NS-90Z Plus). To directly observe their morphology, CM-MSNs were stained with 3% uranyl acetate dihydrate and subsequently imaged by transmission electron microscopy (TEM, Thermo Fisher, Talos F200X).

### 2.9 Validation of membrane purity

To evaluate the purity of the isolated cell membranes, BMSCs were first labeled with DiO (Beyotime, C1038) to stain the plasma membrane and Hoechst (Beyotime, C1011) to stain the nuclei. Fluorescence images were acquired before membrane isolation to confirm the presence of both membrane and nuclear signals. The isolated membrane fraction was resuspended in PBS and subjected to the same staining procedure. Finally, fluorescence microscopy was performed to verify the retention of membrane signals and the absence of nuclear contamination.

### 2.10 Fluorescence colocalization

The cell membranes were marked with the green-fluorescent dye DiO, while the MSNs were labeled with the red-fluorescent dye Rhodamine B (Aladdin, R639790). CM-MSNs were then prepared and imaged using a confocal microscope (Leica, Stellaris). The degree of colocalization was quantitatively analyzed using ImageJ software.

### 2.11 Cellular uptake assay

BMSCs were seeded on glass coverslips and incubated overnight. To visualize nanoparticle uptake, CM-MSNs were labeled by pre-staining the membrane coating with DiO (green) and the MSN cores with Rhodamine B (red). BMSCs were then incubated with the CM-MSNs at 150 μg/mL for 2 h at 37 °C. Cells were then washed with PBS and stained with Hoechst (blue) to label the nuclei. Images were subsequently acquired using a fluorescence microscope.

### 2.12 Elemental analysis

To evaluate the elemental composition and spatial distribution within CM-MSNs, energy-dispersive X-ray spectroscopy (EDS) integrated with TEM was employed. Particular attention was given to elements associated with the cell membrane and MSN core (Si, O, C, N, P). Elemental mapping was conducted to visualize the spatial localization of the elements, and quantitative analysis of the mapped areas was performed to determine their relative enrichment within CM-MSNs.

### 2.13 Coomassie brilliant blue staining

Cell membranes from various differentiation stages and the corresponding CM-MSNs were analyzed by SDS-PAGE. Following electrophoresis, the gels were stained with Coomassie brilliant blue solution (Beyotime, P0003S) on a shaker for 20 min. The gels were then washed and imaged for further analysis.

### 2.14 RNA sequencing and data analysis

Cells were seeded in 6-well plates and cultured in osteogenic differentiation medium. At days 0, 7 and 21, cells were harvested and total RNA was extracted using TRIzol reagent (Vazyme, R401). RNA sequencing was then performed by the Annoroad Co., Ltd. Differential expression analysis was carried out using the DESeq2 R package. Genes with fold change > 2 and p-value < 0.05 were assigned as differentially expressed. The Gene Ontology (GO) enrichment analysis was conducted using the clusterProfiler package in R software.

### 2.15 Proteomic sequencing and data analysis

Cell membranes from different osteogenic stages were isolated and subjected to proteomic sequencing by Majorbio Biomedical Technology Co., Ltd. Proteins with a fold change > 2 and p-value < 0.05 were classified as differentially expressed. These differentially expressed proteins were further annotated and analyzed using GO and the STRING database.

### 2.16 Immunofluorescence staining of BMSCs

BMSCs were seeded in 24-well plates and grouped according to the duration of osteogenic induction: undifferentiated, early osteogenesis, and late osteogenesis. At the end of induction, cells were washed with PBS and fixed with 4% paraformaldehyde, followed by blocking with immunostaining blocking buffer (Beyotime, P0102) for 1 h. The cells were then incubated with the primary antibody overnight at 4 °C. After three washes with PBST, the cells were incubated with fluorescent secondary antibodies and Hoechst staining solution for 1 h. Following three additional washes with PBST, the samples were visualized using a fluorescence microscope.

### 2.17 Biocompatibility assay

BMSCs were co-cultured with a range of MSN concentrations (0, 50, 100, 150, 200, 300, 500, and 1000 μg/mL), and cell viability was assessed after 24 h using the Cell Counting Kit-8 (Beyotime, C0037). In addition, cells were co-cultured with different types and concentrations of nanoparticles for 1, 3, and 7 days, and cell viability was evaluated using the Cell Counting Kit-8 assay. The Calcein AM/PI Cell Viability/Cytotoxicity Assay Kit (Beyotime, C2015M) was further used to assess cell viability and proliferation. The cells were stained with Calcein AM and imaged under a fluorescence microscope.

### 2.18 Oil Red O staining and quantitative analysis

After 14 days of adipogenic differentiation, the cells were washed with PBS and stained with Oil Red O solution at room temperature for 30 min. After staining, the cells were washed again and then imaged under a microscope. To quantify lipid accumulation, the bound dye was eluted with isopropanol (Aladdin, I112011), and the OD of the eluent was measured at 510 nm.

### 2.19 Reverse validation of β-catenin signaling using MSAB

To perform reverse validation of β-catenin signaling, BMSCs were treated with 5 μM MSAB (MedChemExpress, HY-120697). For Western blot and ALP staining experiments, cells were co-cultured with Ctrl, MSN, SC-MSN, EO-MSN, or LO-MSN at 150 μg/mL under osteogenic induction conditions for 7 days. For Oil Red O staining experiments, cells were co-cultured with Ctrl, MSN, SC-MSN, EO-MSN, or LO-MSN at 150 μg/mL under adipogenic induction conditions for 14 days.

### 2.20 *In vivo* retention time and homing effect analysis

MSNs were incubated with indocyanine green (ICG) dye and subjected to ultrasonic treatment in a water bath maintained at 4 °C for 30 min. Subsequently, excess ICG dye was removed through centrifugation to obtain MSNs loaded with ICG (ICG@MSNs). Equivalent amounts of ICG dye, ICG@MSNs, ICG@SC-MSNs, ICG@EO-MSNs and ICG@LO-MSNs were injected into SD rats via the tail vein, and fluorescence distribution was observed at different time points (2 h, 4 h, 8 h, 12 h, 24 h) using an *in vivo* imaging system (IVIS, Xenogen Corporation, Hopkinton, MA). At 8 h after injection, a subset of the rats was euthanized and femoral tissues were collected for IVIS analysis. Fluorescence quantification was performed using Living Image® 4.1 software, with results expressed as average radiance (p/s/cm^2^/sr).

### 2.21 Construction of the OVX model

To simulate postmenopausal osteoporosis, an ovariectomized (OVX) rat model was constructed. SD rats were anesthetized with isoflurane, and the dorsal area was shaved and disinfected. Longitudinal incisions were made bilaterally along the spine, the fallopian tubes were ligated, and the ovaries were excised. The dorsal skin was then sutured layer by layer. One month after surgery, the successful establishment of the OVX rat model was verified using micro-CT imaging and histological staining.

### 2.22 *In vivo* osteoporosis treatment

After successful establishment of the OVX rat model, the rats were assigned to the PBS, MSN, SC-MSN, EO-MSN, and LO-MSN treatment groups (n = 6) and were administered the corresponding formulations via tail vein injection twice per week at a dose of 800 μg per injection. Healthy rats served as the sham group (n = 6). The positive drug control group was established by administering zoledronic acid (ZA) to rats (n = 6). After eight weeks of treatment, the major organs and femurs were harvested for subsequent experiments.

### 2.23 Micro-CT analysis

Femurs were harvested and fixed in 4% paraformaldehyde, then scanned by micro-computed tomography (Micro-CT). Three-dimensional reconstruction of the femurs was performed using CT-Vox software. Bone-related parameters, including bone mineral density (BMD), bone surface-to-total volume ratio (BS/TV), bone volume-to-total volume ratio (BV/TV), trabecular pattern factor (Tb.Pf), cortical bone mineral density (Ct.BMD) and cortical thickness (Ct.Th) were analyzed with CT-Vol software.

### 2.24 Histological staining analysis

Major organs and femurs were collected and fixed in 4% paraformaldehyde. The heart, liver, spleen, lung, and kidney were subjected to hematoxylin and eosin (H&E) staining to assess histological alterations. The femurs were decalcified and then subjected to Masson trichrome staining, H&E staining and immunofluorescence staining. Quantitative analysis of immunofluorescence staining was conducted using ImageJ software.

### 2.25 RT-qPCR analysis

Total RNA was extracted from osteogenic and adipogenic differentiated cells using RNA Total Extraction Reagent (Vazyme, R401). Subsequently, 1 μg RNA was reverse transcribed into complementary DNA (cDNA) using HiScript III All-in-one RT SuperMix (Vazyme, R333). Gene expression was then quantified using the Taq Pro Universal SYBR qPCR Master Mix (Vazyme, Q712) on the 7500 FAST Real-Time PCR System (Applied Biosystems) for 40 cycles. GAPDH was used as the internal control to normalize the expression levels of the genes of interest, and each gene was analyzed in triplicate (n = 3).

### 2.26 Western blotting

Total proteins and membrane proteins were extracted from BMSCs at different stages of osteogenic differentiation for Western blot analysis. Samples containing equal amounts of protein were combined with loading buffer and electrophoresed on 10% SDS-polyacrylamide gels. The proteins were subsequently transferred onto polyvinylidene difluoride membranes (PVDF, Immobilon, IPVH00010) and incubated with primary antibodies, followed by horseradish peroxidase-conjugated secondary antibodies. Subsequently, protein signals were observed and captured using an imaging system. GAPDH was used as the loading control for Western blot analysis of total proteins, whereas Na^+^/K^+^-ATPase was used as the loading control for Western blot analysis of membrane proteins.

### 2.27 Statistical analysis

All data derived from experiments are expressed as the mean ± standard deviation (SD) of values obtained from three or more independent experiments. Statistical analysis was performed using GraphPad Prism version 9.3.1. Statistical significance was determined using the following threshold: ∗*P*< 0.05, ∗∗*P*< 0.01, ∗∗∗*P*< 0.001, ∗∗∗∗*P* < 0.0001, with N.S. indicating *P* > 0.05.

## 3 Results and discussion

### 3.1 BMSCs exhibit significant stage-specific differences during stepwise osteogenesis

**Fig. 1:**
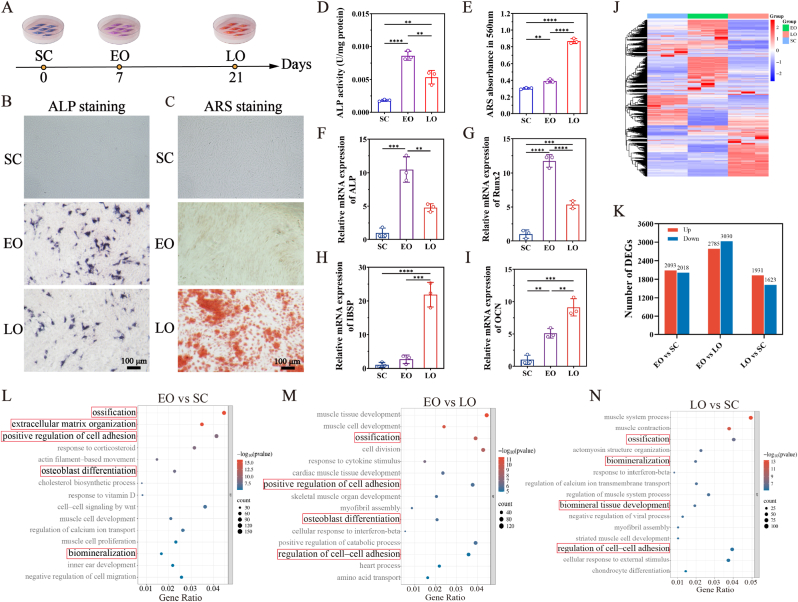
**Stage-specific differences in BMSCs during stepwise osteogenesis.** (A) Timeline of stepwise osteogenic differentiation of BMSCs. Representative ALP staining (B) and ARS staining (C) images of BMSCs in SC, EO, and LO stages. Quantitative analysis of ALP activity (D) and ARS staining (E). Relative mRNA expression of ALP (F), Runx2 (G), IBSP (H), and OCN (I). (J) Clustering heatmap of gene expression among SC, EO and LO. (K) Numbers of differentially expressed genes (DEGs) in pairwise comparisons. (L-N) GO enrichment analysis of DEGs in the indicated comparisons. Data are presented as mean ± SD (n = 3). ∗∗*P*< 0.01, ∗∗∗*P*< 0.001, ∗∗∗∗*P* < 0.0001.

As shown in Fig. 1A, BMSCs were subjected to stepwise osteogenic differentiation by precisely controlling the induction duration: undifferentiated cells were defined as the stem cell stage (SC), whereas cells cultured in osteogenic induction medium for 7 and 21 days were classified as the early osteogenic stage (EO) and the late osteogenic stage (LO), respectively. BMSCs at different osteogenic stages exhibited distinct cellular characteristics, as evidenced by ALP staining and ARS staining (Fig. 1B and C). Cells in the SC stage were negative for both ALP and ARS staining. In contrast, cells in the EO stage displayed the strongest ALP positivity, whereas cells in the LO stage showed markedly enhanced ARS staining. Quantitative analyses further confirmed these stage-specific staining patterns (Fig. 1D and E). During the process of osteogenesis, ALP activity reached its peak at the EO stage and then declined with prolonged differentiation (Fig. S1). ARS staining revealed negligible calcium deposition in the SC and EO stages but became most prominent in the LO stage, reflecting the slow and cumulative nature of calcium deposition. The expression of ALP, Runt-related transcription factor 2 (Runx2), integrin-binding sialoprotein (IBSP), and osteocalcin (OCN) was assessed by RT-qPCR. ALP and Runx2, both recognized as early osteogenic markers, were significantly upregulated after 7 days of osteogenic differentiation (Fig. 1F and G). In contrast, IBSP and OCN, which serve as markers of late osteogenesis, showed significant increases after 21 days (Fig. 1H and I).

RNA sequencing analysis (RNA-seq) was employed to further explore the differences in BMSCs among SC, EO, and LO stages. The clustering heatmap revealed significant variations in gene expression across the three stages (Fig. 1J). Subsequently, we identified differentially expressed genes (DEGs) in pairwise comparisons among the three groups (EO vs SC, EO vs LO, LO vs SC) using a defined statistical threshold (p-value < 0.05, fold change > 2). As illustrated in Fig. 1K and Fig. S2, upregulated and downregulated genes are presented in red and blue, respectively. Gene Ontology (GO) enrichment analysis determined the predominant biological process (BP) associated with these DEGs in each pairwise comparison. The DEGs were primarily enriched in signaling pathways related to osteoblast differentiation, biomineralization, and cell adhesion (Fig. 1L–N). Together, these results indicate that BMSCs undergo dynamic phenotypic and transcriptional changes throughout stepwise osteogenesis.

### 3.2 Preparation and characterization of CM-MSNs

**Fig. 2:**
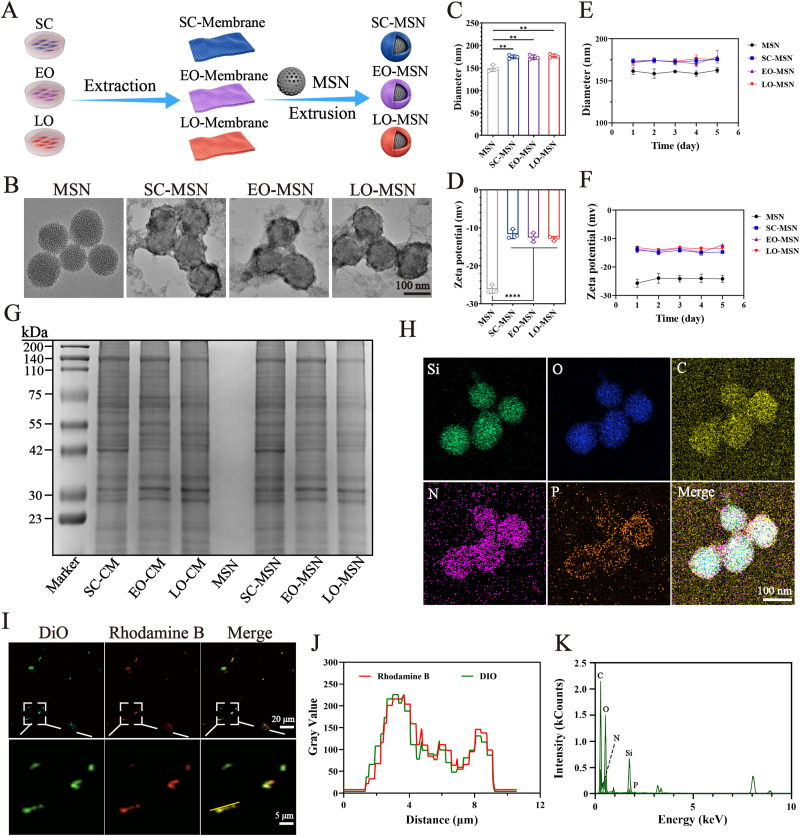
**Construction and characterization of cell membrane-camouflaged MSNs (CM-MSNs).** (A) Experimental scheme for the preparation of CM-MSNs. (B) Representative TEM images of MSNs and CM-MSNs. Hydrodynamic diameter (C) and zeta potential (D) of different nanoparticles. (E-F) Changes in diameter and zeta potential of different nanoparticles in PBS at 4 °C over 5 days. (G) SDS-PAGE profiles of CMs and the corresponding CM-MSNs. (H) Elemental mapping of CM-MSN demonstrated distribution of silicon, oxygen, carbon, nitrogen and phosphorus. (I) Confocal fluorescence images showed colocalization between DiO-labeled cell membranes and Rhodamine B-labeled MSN cores. (J) Fluorescence colocalization curves of CMs and MSNs. (K) EDS spectrum of CM-MSNs. Data are presented as mean ± SD (n = 3). ∗∗*P*< 0.01, ∗∗∗∗*P* < 0.0001.

As illustrated in Fig. 2A, cell membranes (CMs) were isolated through cell lysis followed by differential centrifugation and ultracentrifugation. The isolated cell membranes showed high purity with no detectable nuclear contamination and retained a characteristic bilayer vesicular morphology (Fig. S3A and B). MSNs were synthesized using a classical method as described in Ref. [32]. Then, the synthesized MSNs were co-extruded with three types of cell membranes from different stages of osteogenesis to form three types of CM-MSNs: stem cell membrane-camouflaged MSNs (SC-MSNs), early osteogenesis cell membrane-camouflaged MSNs (EO-MSNs), and late osteogenesis cell membrane-camouflaged MSNs (LO-MSNs). The ratio of membrane to nanoparticles was determined based on previously published research, employing a mass ratio of membrane to nanoparticles of 5:2 [33]. TEM images showed that all three CM-MSNs displayed a typical core-shell structure with relatively uniform particle size, confirming successful membrane coating (Fig. 2B). In alignment with the TEM findings, dynamic light scattering (DLS) showed that the hydrodynamic diameters of the CM-MSNs were all approximately 170 nm (Fig. 2C). In addition, the zeta potential of CM-MSNs was approximately −12 mV, which was slightly higher than that of bare MSNs (Fig. 2D). CM-MSNs remained stable in PBS at 4 °C over 5 days based on DLS measurements (Fig. 2E and F). In contrast, the isolated cell membranes exhibited rapid aggregation when dispersed in PBS at 4 °C (Fig. S4A and B). These results suggest that MSNs serve as a stabilizing core can effectively enhance the dispersibility and stability of CM-MSNs. To evaluate potential protein loss during the extrusion process, SDS-PAGE followed by Coomassie Brilliant Blue staining was performed (Fig. 2G). The similar electrophoretic band profiles of CMs and corresponding CM-MSNs verified the successful membrane coating and the integrity of membrane proteins after extrusion. Elemental mapping showed Si and O signals were localized to the nanoparticle core while C, N and P signals were distributed on the outer region, forming a clear core-shell pattern (Fig. 2H). The corresponding EDS spectrum further supported the successful coating of cell membranes onto the MSN core (Fig. 2K). In addition, confocal fluorescence imaging revealed clear colocalization between the DiO-labeled cell membranes and Rhodamine B-labeled MSN cores (Fig. 2I and J), which provided additional evidence for membrane coating. Furthermore, as shown in Fig. S5, cellular uptake analysis demonstrated that all three CM-MSNs were capable of binding to BMSCs and being internalized.

### 3.3 Proteomic analysis revealed the protein composition and potential functions of the stage-specific membranes

**Fig. 3:**
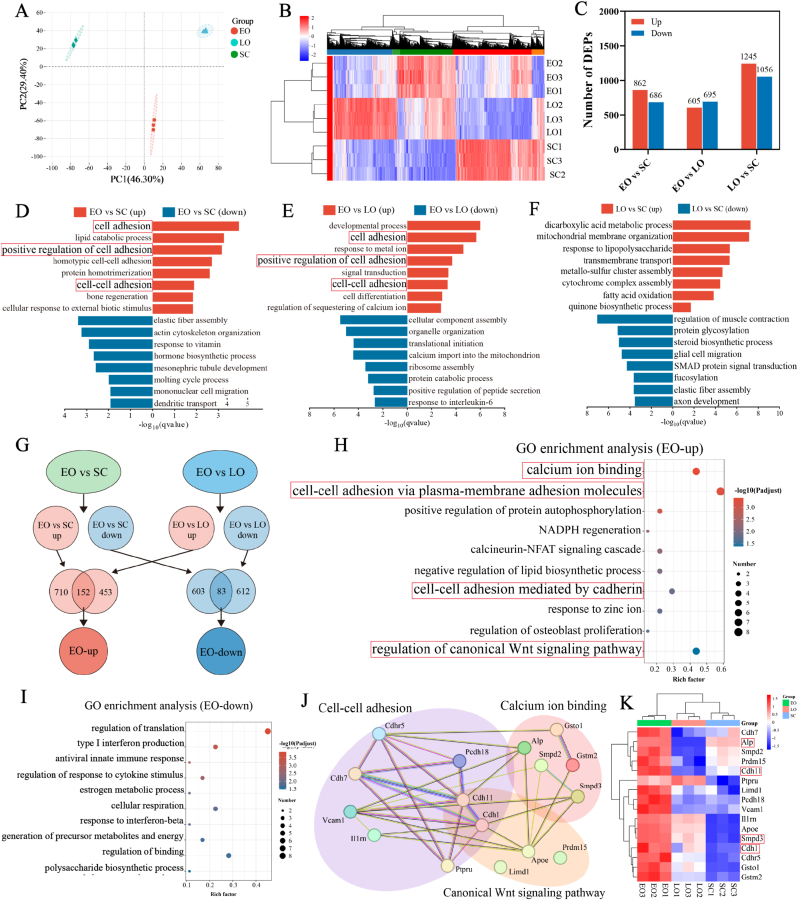
**Proteomic analysis of stage-specific membrane.** (A) PCA plot of SC, EO and LO groups. (B) Clustering heatmap of differentially expressed proteins (DEPs) among the three groups. (C) Numbers of upregulated and downregulated DEPs in each pairwise comparison. (D-F) GO enrichment analysis of upregulated and downregulated DEPs in different comparisons. (G) Workflow for obtaining the target protein set used in subsequent analysis. (H) GO enrichment analysis of the EO-up protein set, signaling pathways related to cell adhesion were highlighted with red boxes. (I) GO enrichment analysis of the EO-down protein set. (J) PPI network analysis of proteins from marked signaling pathways. (K) Heatmap of representative proteins identified by proteomic analysis.

Proteomic analysis was performed on cell membranes isolated from BMSCs at the SC, EO, and LO stages to identify differentially expressed proteins (DEPs). Principal component analysis (PCA) of the proteomic data showed a clear separation among the three groups (Fig. 3A). The clustering heatmap also revealed marked differences in protein abundance across the three stages (Fig. 3B). The numbers of upregulated and downregulated DEPs identified in each pairwise comparison are presented in Fig. 3C (p-value < 0.05, fold change > 2).

These DEPs were subsequently subjected to Gene Ontology (GO) enrichment analysis. Notably, compared with the SC and LO groups, DEPs in the EO group were primarily enriched in signaling pathways related to cell adhesion (Fig. 3D and E). In contrast, in the comparison between the LO and SC groups, the signaling pathways did not show this characteristic enrichment pattern (Fig. 3F). To further define the protein features of the EO stage, we focused on proteins that were consistently upregulated (EO-up) or downregulated (EO-down) in EO relative to both SC and LO (Fig. 3G). GO enrichment analysis showed that the EO-up protein set was mainly associated with cadherin-mediated cell-cell adhesion, calcium ion binding, and regulation of the canonical Wnt signaling pathway (Fig. 3H). In contrast, the EO-down protein set was mainly enriched in processes related to translational regulation, immune responses, and responses to external stimuli (Fig. 3I). Based on protein–protein interaction (PPI) network analysis of proteins from marked GO enriched pathways, proteins were divided into three primary functional categories: cell-cell adhesion, calcium ion binding and canonical Wnt signaling pathway (Fig. 3J). The heatmap in Fig. 3K showed the expression of representative proteins from these enriched functional categories. Membrane-associated phosphatases, such as ALP and Smpd3, are known to promote calcium deposition and biomineralization [22]. In addition, cadherins play critical roles in mediating cell adhesion and regulating proliferation, differentiation, and cell fate [34,35]. Based on these findings and previous studies, representative phosphatases and cadherins were highlighted in Fig. 3K.

**Fig. 4:**
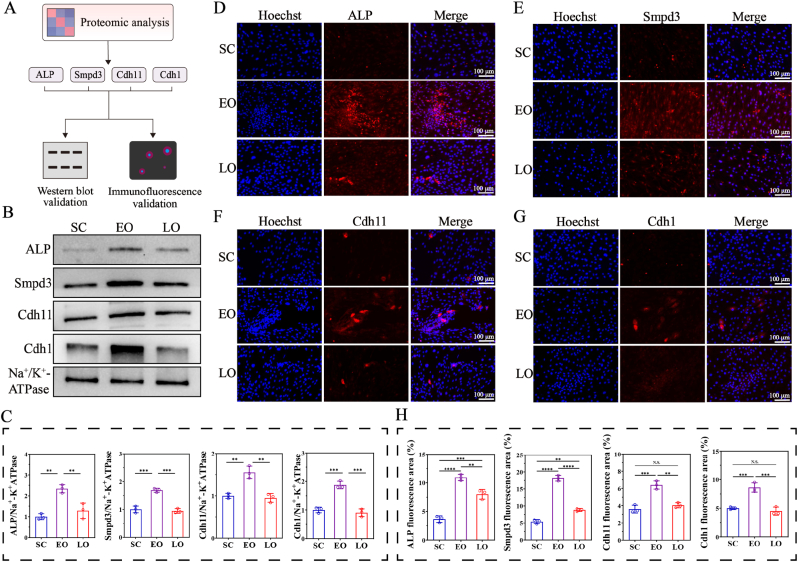
**Validation of representative proteins in BMSCs at different osteogenic stages.** (A) Schematic diagram of the workflow for identifying and validating representative proteins through proteomic and molecular biology experiments. (B) Western blot analysis of ALP, Smpd3, Cdh11, and Cdh1 in cell membrane from different stages. (C) Quantitative analysis of Western blot results. Representative immunofluorescence images of ALP (D), Smpd3 (E), Cdh11 (F), and Cdh1 (G). (H) Quantitative analysis of immunofluorescence staining. Data are presented as mean ± SD (n = 3). N.S., no significant difference, ∗∗*P*< 0.01, ∗∗∗*P*< 0.001, ∗∗∗∗*P* < 0.0001.

Western blot and immunofluorescence staining were employed to validate the expression of these aforementioned representative proteins in BMSCs at SC, EO and LO stages (Fig. 4A). The expression of ALP, Smpd3, Cdh11 and Cdh1 on the cell membrane was assessed through Western blot firstly (Fig. 4B). Combining quantitative analysis further confirmed that these characteristic proteins were significantly enriched in the EO group (Fig. 4C). Western blot analysis was also performed to examine the retention of representative functional proteins after membrane coating. As shown in Fig. S6, ALP and Cdh11 signals were clearly detected in EO-MSNs, confirming that these key membrane proteins were retained after extrusion. The immunofluorescence staining exhibited a trend consistent with the results of Western blot (Fig. 4D–H). The EO group showed prominently higher fluorescence intensities than both SC and LO group. In addition, isolated membranes from different stages were cultured in osteogenic differentiation medium for 21 days, followed by ARS staining to evaluate their ability to promote mineral deposition (Fig. S7). Consistent with the higher phosphatase expression in EO membranes, the EO group exhibited the strongest ARS staining, suggesting a greater capacity to promote mineralization.

Together, these proteomic and experimental data indicate that BMSCs at the EO stage exhibit a distinct membrane protein profile related to phosphatases and adhesion molecules. These stage-dependent differences in protein composition and expression are likely to substantially influence membrane properties and biological functions.

### 3.4 *In vitro* evaluation of the biocompatibility and differentiation-regulating effects of CM-MSNs on BMSCs

The biocompatibility of MSNs at different concentrations was evaluated via the CCK-8 assay. As shown in Fig. S8A, after 24 h of co-culturing, MSN concentrations ranging from 0 to 150 μg/mL did not exhibit significant cytotoxicity. However, cell viability significantly decreased when the concentration reached 200 μg/mL. To further investigate whether different cell membrane coatings affect the biocompatibility of the MSN, BMSCs were co-cultured with different types of nanoparticles at concentrations of 50, 100, and 150 μg/mL. Cell viability was assessed by CCK-8 assay at 1, 3, and 7 days (Fig. S8B–D). No significant differences in cell viability were observed among the groups at any time point. Live cell staining further showed similar cell densities across the different groups, indicating that CM-MSNs did not noticeably affect cell proliferation (Fig. S8E). To determine the optimal working concentration for subsequent experiments, a concentration-gradient study was performed to evaluate the effects of CM-MSNs on BMSC osteogenic differentiation (Fig. S9). In the absence of significant cytotoxicity, 150 μg/mL produced the most pronounced pro-osteogenic effect and was therefore selected for subsequent *in vitro* experiments. In addition, a control experiment using isolated cell membranes was performed to compare the effects of CMs and CM-MSNs on the osteogenic differentiation of BMSCs (Fig. S10). The results showed that cell membranes alone exerted a significantly weaker effect on osteogenic differentiation compared with CM-MSN. This may be attributed to the absence of the MSN core, which compromises structural stability and limits the effective presentation of membrane proteins. Taken together, these results support the use of 150 μg/mL CM-MSNs as the working concentration for subsequent *in vitro* studies.

**Fig. 5:**
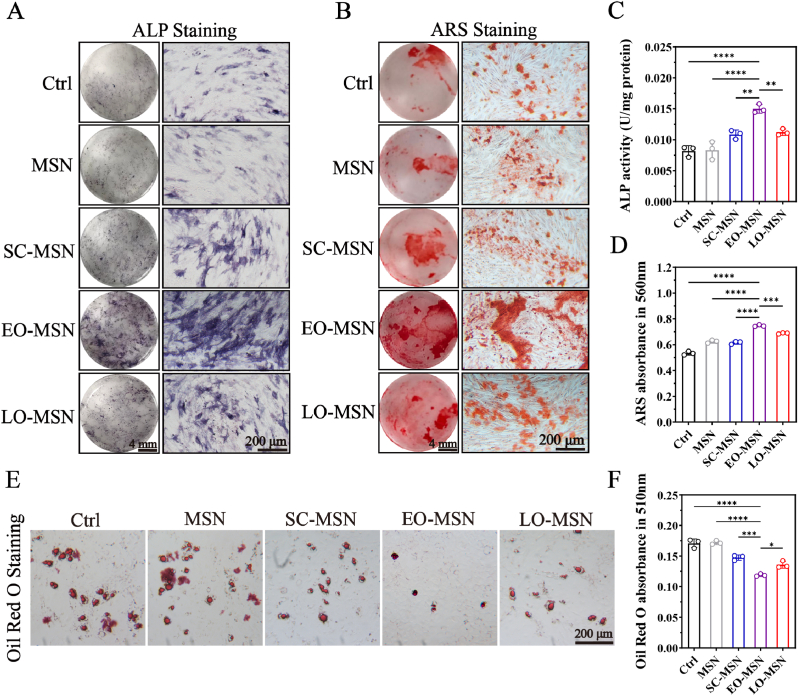
***In vitro*****differentiation-regulating effects of CM-MSNs on BMSCs.** Representative ALP staining (A) and ARS staining (B) images of different groups. Quantitative analysis of ALP activity (C) and ARS staining (D). Representative Oil Red O staining images (E) and quantitative analysis (F) of different groups. Data are presented as mean ± SD (n = 3). ∗*P*< 0.05, ∗∗*P*< 0.01, ∗∗∗*P*< 0.001, ∗∗∗∗*P* < 0.0001.

To investigate the regulatory effects of CM-MSNs on BMSC differentiation, cells were co-cultured with different CM-MSNs under osteogenic or adipogenic induction conditions. After 7 days of osteogenic induction, ALP staining and quantitative analysis were performed (Fig. 5Aand C). Compared with the Ctrl and MSN groups, CM-MSN treatment enhanced ALP expression, with EO-MSN showing the strongest effect. After 21 days of osteogenic induction, ARS staining and quantitative analysis showed that CM-MSN, particularly EO-MSN, significantly enhanced calcium deposition (Fig. 5Band D). The osteogenic and adipogenic differentiation of BMSCs exhibit an antagonistic relationship [[36], [37], [38]]. Adipogenic differentiation was further evaluated after 14 days of induction. Oil Red O staining and quantitative analysis showed that CM-MSN effectively reduced lipid droplet formation, with EO-MSN exhibiting the strongest inhibitory effect among all groups (Fig. 5E and F).

### 3.5 CM-MSNs regulated the balance between osteogenesis and adipogenesis via the Wnt/β-catenin signaling pathway

**Fig. 6:**
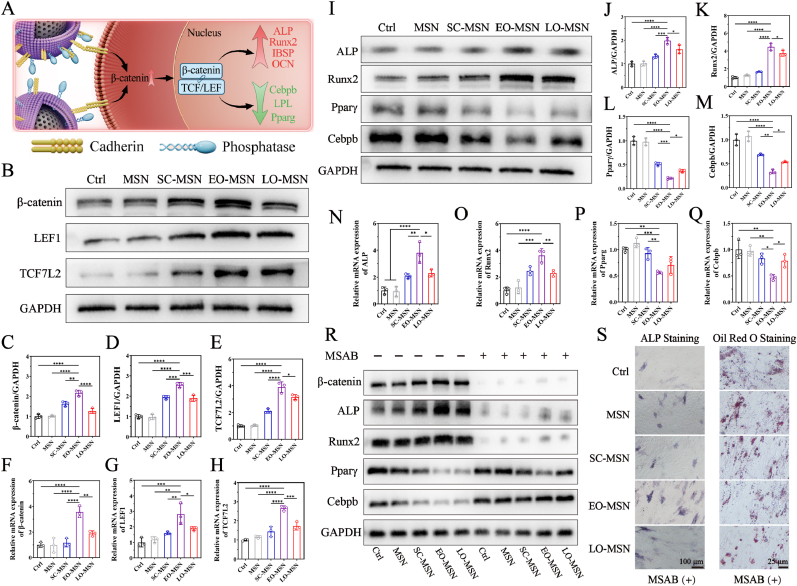
**Biological mechanism** by which **CM-MSNs regulate the balance of osteogenesis and adipogenesis**. (A) The schematic diagram shows the possible mechanisms of EO-MSNs to affect osteogenesis and adipogenesis. (B) Western blot assay of β-catenin, LEF1 and TCF7L2 in different groups. (C-E) Quantitative analysis of Western blot (β-catenin, LEF1 and TCF7L2). (F-H) RT-qPCR evaluates marker genes of the Wnt/β-catenin signaling pathway (β-catenin, LEF1 and TCF7L2). (I) Western blot assay of osteogenic and adipogenic differentiation-related markers in different groups. (J-M) Quantitative analysis of Western blot (ALP, Runx2, Pparγ and Cebpb). (N-Q) RT-qPCR evaluates marker genes of osteogenesis and adipogenesis (ALP, Runx2, Pparg and Cebpb). (R) Western blot assay of β-catenin, ALP, Runx2, Pparγ, and Cebpb of different groups in the absence (−) or presence (+) of MSAB. (S) Representative ALP staining and Oil Red O staining images of different groups treated with MSAB. Data are presented as mean ± SD (n = 3). ∗*P*< 0.05, ∗∗*P*< 0.01, ∗∗∗*P*< 0.001, ∗∗∗∗*P* < 0.0001.

β-catenin is an intracellular glycoprotein with essential functions, which forms a complex with cadherin to be involved in intercellular interaction [39]. Zhu et al. demonstrated that exogenous cadherin can enhance osteogenesis via activation of the Wnt/β-catenin signaling pathway [40]. Building on these findings, Fig. 6A reveals the potential mechanism by which CM-MSNs facilitate osteogenesis and inhibit adipogenesis. BMSCs were co-cultured with different membrane-camouflaged MSNs for 7 days, and β-catenin expression in each group was detected through Western blot and RT-qPCR (Fig. 6B, C, F). The results demonstrated that CM-MSNs treatment increased β-catenin expression, with EO-MSNs showing the strongest effect. Subsequently, the expression of downstream markers in the Wnt/β-catenin signaling pathway were also assessed by Western blot and RT-qPCR (Fig. 6B, D, E, G, H). Both LEF1 and TCF7L2 were significantly upregulated in the EO-MSN group. To further elucidate whether the activation of Wnt/β-catenin signaling affected BMSC differentiation, cells were co-cultured with different CM-MSNs under osteogenic or adipogenic induction conditions. Under osteogenic induction, the expression of ALP and Runx2 was evaluated after 7 days (Fig. 6I, J, K, N, O). Compared with the Ctrl and MSN groups, CM-MSNs treatment significantly increased the expression of osteogenic markers, with EO-MSNs showing the strongest effect. Under adipogenic induction, the expression of adipogenic markers was assessed after 14 days (Fig. 6I, L, M, P, Q). CM-MSNs treatment significantly suppressed adipogenic differentiation, particularly in the EO-MSN group.

To further validate the underlying mechanism, the β-catenin inhibitor MSAB was used to suppress β-catenin signaling and evaluate its effects. As shown in Fig. 6R and Fig. S11A–E, Western blot analysis demonstrated that MSAB effectively inhibited β-catenin signaling, leading to decreased expression of osteogenic related proteins (ALP and Runx2), while increasing the expression of adipogenic related proteins (Pparγ and Cebpb). Consistently, ALP staining and quantitative analysis revealed that the pro-osteogenic effect of the nanoparticles was significantly attenuated following MSAB treatment, whereas Oil Red O staining and its quantification indicated enhanced adipogenic differentiation (Fig. 6S and Fig. S12A and B).

Together, these results demonstrated that CM-MSNs, particularly EO-MSNs, activate the Wnt/β-catenin signaling pathway via enriched cadherins on their membrane surface, thereby regulating the differentiation of BMSCs.

### 3.6 CM-MSNs prolonged circulation time and accelerated bone-specific accumulation *in vivo*

**Fig. 7:**
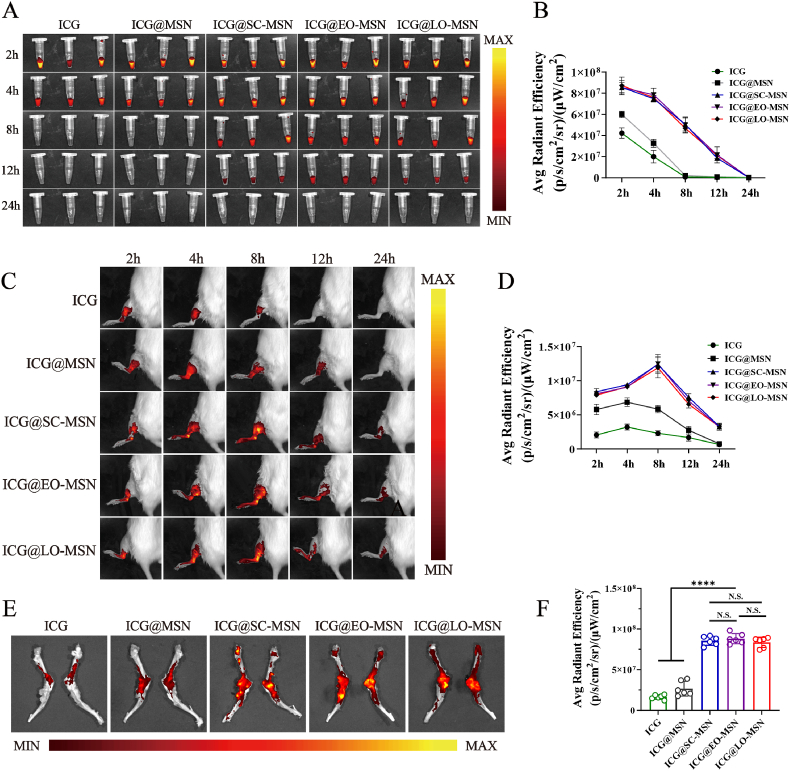
***In vivo*****long circulation and bone homing effect of CM-MSN.** (A) IVIS images of blood samples collected at different time points after injection. (B) Fluorescence intensity curve of blood at different time points. (C) IVIS images of rat hindlimbs at different time points after injection. (D) The curve of fluorescence intensity changes in rat hindlimbs over time. IVIS images (E) and fluorescence intensities (F) of femoral tissue after injection for 8h. Data are presented as mean ± SD (n = 6). N.S., no significant difference, ∗∗∗∗*P* < 0.0001.

Cell membrane camouflaging can grant nanoparticles immune escape properties and prolonged circulation capabilities *in vivo* [41,42]. To evaluate the circulation behavior of CM-MSNs in rats, MSNs were labeled with indocyanine green (ICG), and equal amounts of free ICG, ICG@MSN, ICG@SC-MSN, ICG@EO-MSN, and ICG@LO-MSN were administered via tail vein injection. Blood samples were collected at different time points, and fluorescence intensity was measured using an *in vivo* imaging system (IVIS). As shown in Fig. 7A and B, fluorescence in the free ICG and ICG@MSN groups decreased rapidly and vanished after 8 h. In contrast, owing to the biomimetic membrane coating, the ICG@SC-MSN, ICG@EO-MSN, and ICG@LO-MSN groups showed markedly slower clearance from the circulation, with detectable fluorescence still present at 12 h.

The homing effect of stem cells has been well established [43,44]. To investigate whether membrane coating still possess this function, equal amounts of different nanoparticles were injected into rats through the tail vein, and fluorescence signals in the hindlimbs were monitored by IVIS at different time points (Fig. 7C and D). Fluorescence in the free ICG group declined rapidly and disappeared by 12 h. Compared with free ICG, the ICG@MSN group showed slightly prolonged fluorescence retention, likely due to nanoparticle-mediated protection of the dye during circulation. Notably, ICG@SC-MSN, ICG@EO-MSN, and ICG@LO-MSN groups exhibited significantly higher fluorescence intensity in the hindlimbs than the free ICG and ICG@MSN groups. The fluorescence signal peaked at 8 h post-injection and remained detectable at 24 h. At 8 h after injection, femoral tissues were harvested for fluorescence analysis. As shown in Fig. 7E and F, fluorescence intensities in the femurs from the ICG and ICG@MSN groups were significantly lower than those in the other three CM-MSN groups. These results demonstrate that cell membranes derived from different osteogenic stages markedly enhance the homing effect of MSN toward bone tissue. Notably, SC-MSN, EO-MSN, and LO-MSN exhibited roughly equivalent homing effects, suggesting that subsequent therapeutic differences are unlikely to be driven by targeting magnitude, but rather by the stage-dependent bioactive surface proteins on their membrane coating.

### 3.7 Anti-osteoporotic effects of CM-MSNs in OVX rats

**Fig. 8:**
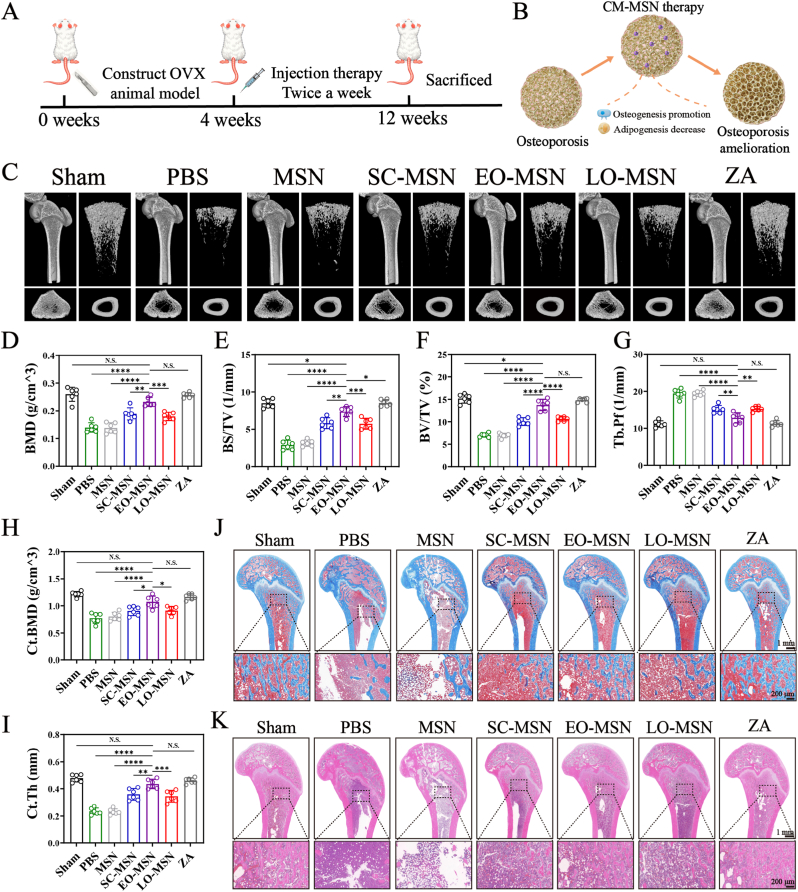
**CM-MSNs ameliorate the structure of the femur.** (A) Timeline of *in vivo* treatment experiments. (B) Schematic diagram of osteoporosis amelioration. (C) Micro-CT images of the femur from different groups. Quantitative analysis of cancellous bone parameters, including BMD (D), BS/TV (E), BV/TV (F), and Tb.Pf (G). Quantitative analysis of cortical bone parameters, including Ct.BMD (H) and Ct.Th (I). (J) Representative Masson trichrome staining images of the femur from different groups. (K) Representative H&E staining images of the femur from different groups. Data are presented as mean ± SD (n = 6). N.S., no significant difference, ∗*P*< 0.05, ∗∗*P*< 0.01, ∗∗∗*P*< 0.001, ∗∗∗∗*P* < 0.0001.

An ovariectomized (OVX) rat model was established to mimic postmenopausal osteoporosis (OP). The treatment schedule is shown in Fig. 8A. At the end of the 8-week treatment period, the rats were sacrificed and tissues were collected for subsequent analyses. As illustrated in Fig. 8B, CM-MSNs were designed to alleviate osteoporosis by promoting osteogenesis while suppressing adipogenesis. Micro-CT analysis of femurs was performed to evaluate the degree of OP amelioration (Fig. 8C). Compared with the other treatment groups, the EO-MSN group exhibited denser and more intact trabecular structures, most closely resembling those of the sham and zoledronic acid (ZA) groups. Quantitative analysis further showed that EO-MSN treatment significantly improved multiple bone parameters, including BMD, BS/TV, BV/TV, Tb.Pf, Ct.BMD, and Ct.Th (Fig. 8D–I). These results indicate that EO-MSN markedly alleviated OVX-induced deterioration of femoral bone structure. To further assess the anti-osteoporotic effects of CM-MSNs, femoral tissues were analyzed by Masson's trichrome staining and H&E staining. Masson trichrome staining revealed clear differences in trabecular architecture and matrix deposition among the groups (Fig. 8J). Compared with the sham group, the OVX group showed pronounced trabecular loss, enlarged marrow spaces, and reduced blue-stained bone matrix. In contrast, EO-MSN treatment resulted in more abundant and continuous blue-stained bone matrix, together with a denser and more intact trabecular network, yielding an overall morphology closer to that of the sham group. Similarly, H&E staining showed that the EO-MSN group exhibited more abundant trabecular structures than the other treatment groups, closely resembling the sham and ZA groups (Fig. 8K).

**Fig. 9:**
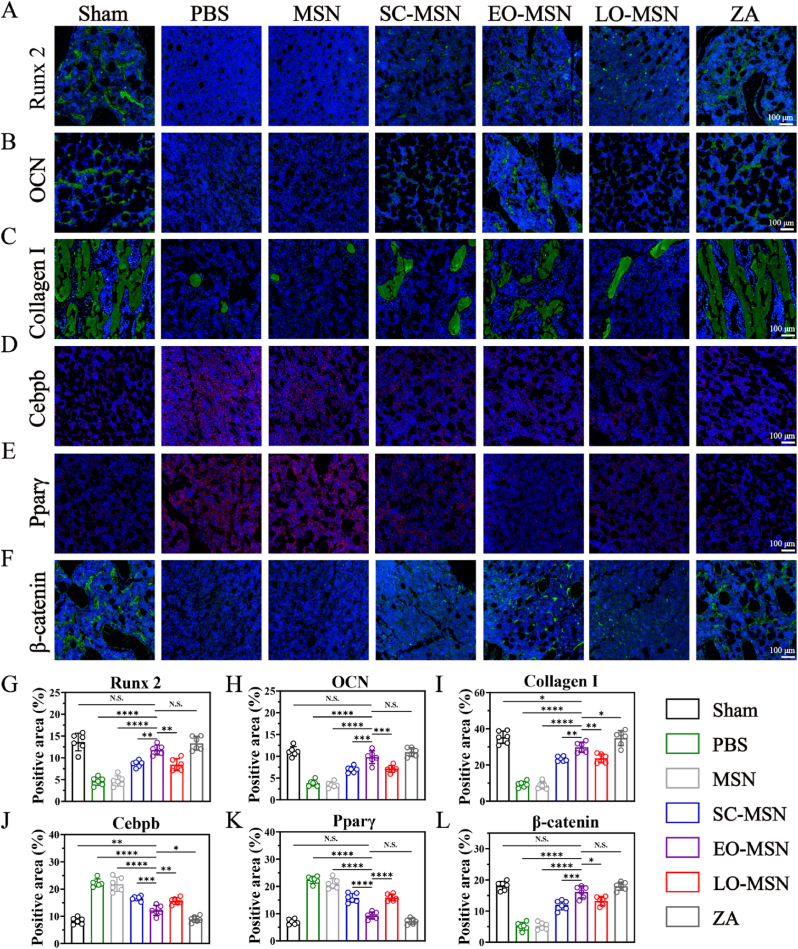
**Immunofluorescence staining of bone tissue.** (A-C) Representative immunofluorescence staining images of osteogenic markers in femur from different groups. (D-E) Representative Immunofluorescence staining images of adipogenic markers in femur from different groups. (F) Representative Immunofluorescence staining images of β-catenin in femur from different groups. Quantitative analysis of Runx2 (G), OCN (H), Collagen I (I), Cebpb (J), Pparγ (K), and β-catenin (L) positive areas. Data are presented as mean ± SD (n = 6). N.S., no significant difference, ∗*P*< 0.05, ∗∗*P*< 0.01, ∗∗∗*P*< 0.001, ∗∗∗∗*P* < 0.0001.

Immunofluorescence staining was further performed to assess the expression of osteogenic markers, including Runx2, OCN, and collagen I (Fig. 9A–C). The EO-MSN group showed stronger positive signals for all three markers, closely resembling the sham and ZA groups. Quantitative analysis further confirmed the significant osteogenic effect of EO-MSN in *vivo* (Fig. 9G–I). In addition, adipogenic markers in bone tissue were evaluated by immunofluorescence staining and quantitative analysis (Fig. 9D, E, J, K). The EO-MSN group exhibited markedly reduced adipogenic marker signals, further supporting its inhibitory effect on adipogenic differentiation *in vivo*. Furthermore, β-catenin signal in bone tissue was also increased following EO-MSN treatment (Fig. 9Fand L). This result is consistent with the *in vitro* findings and provides additional evidence that EO-MSN effectively activates the Wnt/β-catenin signaling pathway *in vivo*.

To evaluate the *in vivo* biosafety of CM-MSNs, body weight was recorded weekly throughout the treatment process. No significant differences in body weight were observed among the groups (Fig. S13). H&E staining of the heart, liver, spleen, lung, and kidney showed no obvious histological abnormalities (Fig. S14). Serum IL-6 and TNF-α levels were measured by ELISA to assess systemic inflammatory responses. As shown in Fig. S15A and B, no significant differences were observed among the groups, indicating that the treatments did not induce detectable systemic inflammation. Moreover, serum biochemical analysis showed no significant differences among the groups after different treatments (Fig. S16). Together, these findings support the excellent *in vivo* biosafety of CM-MSNs.

## 4 Conclusion

This study investigated stage-specific differences in BMSC membranes during stepwise osteogenesis. Proteomic analysis and molecular biological experiments revealed dynamic alterations in membrane protein composition, with phosphatases and cadherins markedly enriched at the early osteogenic stage. Based on these findings, we constructed cell membrane-camouflaged mesoporous silica nanoparticles (CM-MSNs) for anti-osteoporotic therapy, among which EO-MSNs exhibited the most pronounced therapeutic efficacy. BMSC-derived membranes endowed the nanoparticles with bone homing effect, enabling EO-MSNs to preferentially accumulate in bone tissue. Phosphatases provided ideal nucleation sites for calcium deposition and decreased calcium loss in bone tissue. Meanwhile, cadherins present on cell membranes regulated osteogenic and adipogenic differentiation via the Wnt/β-catenin signaling pathway. Consequently, our work suggests that stepwise osteogenesis cell membranes may serve as a distinctive and effective biofunctional material for modulating the osteoblast-adipocyte balance in osteoporotic microenvironment, offering a novel perspective for OP treatment.

## CRediT authorship contribution statement

**Kehan Cai:** Formal analysis, Investigation, Visualization, Writing – original draft. **Zhe Fan:** Formal analysis, Software, Validation. **Jun Tan:** Formal analysis, Investigation. **Wei Cai:** Data curation, Validation. **Minghang Zhang:** Investigation. **Haojie Tang:** Resources. **Jianzhong Xu:** Supervision. **Han Lu:** Formal analysis. **Wei He:** Conceptualization. **Qian Xu:** Conceptualization, Writing – review & editing. **Yazhou Chen:** Conceptualization, Methodology, Project administration, Writing – review & editing. **Tao Chen:** Funding acquisition, Resources, Supervision, Writing – review & editing.

## Ethics approval and consent to participate

All animal experiments were conducted following protocols approved by the Institutional Animal Care and Use Committee of Zhengzhou University (approval number: ZZU-LAC20240531 [13]).

## Declaration of competing interest

The authors declare no conflicts of interest.

## Data Availability

All data generated or analyzed during this work are available upon request from the corresponding authors.

## References

[1] Chen J., Hao Z., Li H., Wang J., Chen T., Wang Y., Shi G., Wang J., Wang Z., Zhang Z., Li J. (2024). Osteoporotic osseointegration: therapeutic hallmarks and engineering strategies. Theranostics.

[2] Wang J., Chen R., Ren B., Feng Q., Li B., Hao Z., Chen T., Hu Y., Huang Y., Zhang Q., Wang Y., Huang J., Li J. (2023). A novel PTH-related peptide combined with 3D printed macroporous titanium alloy scaffold enhances osteoporotic osseointegration. Adv. Healthcare Mater..

[3] Fischer V., Haffner-Luntzer M. (2022). Interaction between bone and immune cells: implications for postmenopausal osteoporosis. Semin. Cell Dev. Biol..

[4] Luo Z.-H., Ma J.-X., Zhang W., Tian A.-X., Gong S.-W., Li Y., Lai Y.-X., Ma X.-L. (2023). Alterations in the microenvironment and the effects produced of TRPV5 in osteoporosis. J. Transl. Med..

[5] Meng S., Liu Q., Dai R., Wang Y., Chen L., Wu S., Li J., Zhang J., Gao M., Kang W., Zheng Z., Wu H., Zhang R. (2025). Development of a novel macroscopic regulation and microscopic intervention mode nanosystem for osteoporosis treatment. Mater. Today Bio.

[6] Zhang Q., Hu S., Wu J., Sun P., Zhang Q., Wang Y., Zhao Q., Han T., Qin L., Zhang Q. (2023). Nystose attenuates bone loss and promotes BMSCs differentiation to osteoblasts through BMP and wnt/*β*-catenin pathway in ovariectomized mice. Food Sci. Hum. Wellness.

[7] Reid I.R., Billington E.O. (2022). Drug therapy for osteoporosis in older adults. Lancet.

[8] Hao Z., Feng Q., Wang Y., Wang Y., Li H., Hu Y., Chen T., Wang J., Chen R., Lv X., Yang Z., Chen J., Guo X., Li J. (2024). A parathyroid hormone related supramolecular peptide for multi-functionalized osteoregeneration. Bioact. Mater..

[9] Chen T., Wang Y., Hao Z., Hu Y., Li J. (2021). Parathyroid hormone and its related peptides in bone metabolism. Biochem. Pharmacol..

[10] Lei C., Song J., Li S., Zhu Y., Liu M., Wan M., Mu Z., Tay F.R., Niu L. (2023). Advances in materials-based therapeutic strategies against osteoporosis. Biomaterials.

[11] Hu Y., Zhang H., Wang S., Cao L., Zhou F., Jing Y., Su J. (2023). Bone/cartilage organoid on-chip: construction strategy and application. Bioact. Mater..

[12] Jiang Y., Zhang P., Zhang X., Lv L., Zhou Y. (2021). Advances in mesenchymal stem cell transplantation for the treatment of osteoporosis. Cell Prolif..

[13] Phetfong J., Sanvoranart T., Nartprayut K., Nimsanor N., Seenprachawong K., Prachayasittikul V., Supokawej A. (2016). Osteoporosis: the current status of mesenchymal stem cell-based therapy. Cell. Mol. Biol. Lett..

[14] Chen T., Yang T., Zhang W., Shao J. (2021). The therapeutic potential of mesenchymal stem cells in treating osteoporosis. Biol. Res..

[15] Park S., Rahaman K.A., Kim Y.-C., Jeon H., Han H.-S. (2024). Fostering tissue engineering and regenerative medicine to treat musculoskeletal disorders in bone and muscle. Bioact. Mater..

[16] Zhao Y., Li R., Han Y., Shi C., Lee K., Nie G., Chen Y. (2026). On-demand cancer immunotherapy via single-cell encapsulation of synthetic circuit-engineered cells. Sci. Adv..

[17] Zhao Y., Chuai Y., Fu K., Han Y., Gao N., Nie G., Chen Y. (2026). Synthetic biology integrated with material science paves the way for next-generation smart cell therapies. Mol. Ther. Oncol..

[18] Chen B.-Q., Zhao Y., Zhang Y., Pan Y.-J., Xia H.-Y., Kankala R.K., Wang S.-B., Liu G., Chen A.-Z. (2023). Immune-regulating camouflaged nanoplatforms: a promising strategy to improve cancer nano-immunotherapy. Bioact. Mater..

[19] Liu H., Li Y., Wang Y., Zhang L., Liang X., Gao C., Yang Y. (2025). Red blood cells-derived components as biomimetic functional materials: matching versatile delivery strategies based on structure and function. Bioact. Mater..

[20] Song W., Jia P., Ren Y., Xue J., Zhou B., Xu X., Shan Y., Deng J., Zhou Q. (2023). Engineering white blood cell membrane-camouflaged nanocarriers for inflammation-related therapeutics. Bioact. Mater..

[21] Liao J., Lu L., Chu X., Xiong Y., Zhou W., Cao F., Cheng P., Shahbazi M.-A., Liu G., Mi B. (2024). Cell membrane coated nanoparticles: cutting-edge drug delivery systems for osteoporosis therapy. Nanoscale.

[22] Hatano E., Akhter N., Anada R., Ono M., Oohashi T., Kuboki T., Kamioka H., Okada M., Matsumoto T., Hara E.S. (2024). The cell membrane as biofunctional material for accelerated bone repair. Acta Biomater..

[23] Yang L.-Y., Huang M., Zheng X.-L., Liu M.-X., Wu Q.-B., Fan X.-X. (2026). Biomimetic universal CAR-mesenchymal stem cell nanohybrids for anti-tumor therapy. Bioact. Mater..

[24] Chen Y., Qin H., Li N., Wei Y., Lin Y., Deng R., Ding H., Lv Y., Ma T., Li R., Xiong C., Zheng G., Chen H., Shi J., Zhao Y., Zhao R., Nie G. (2025). Neoadjuvant chemotherapy by liposomal doxorubicin boosts immune protection of tumor membrane antigens-based nanovaccine. Cell Rep. Med..

[25] He Z., Hu P., Li Z., Mao K., Zheng J., Yang C.-Y., Luo Y., Yang J., Cao Z., Lu J., Luo X., Tong S., He Z., Kim K., Liu Y., Sun X., Zhao L., Pan Y., Cao Y., Wang Y., Wang X. (2025). Self-assembled hybrid hydrogel microspheres create a bone marrow-mimicking niche for bone regeneration. Bioact. Mater..

[26] van de Peppel J., Strini T., Tilburg J., Westerhoff H., van Wijnen A.J., van Leeuwen J.P. (2017). Identification of three early phases of cell-fate determination during osteogenic and adipogenic differentiation by transcription factor dynamics. Stem Cell Rep..

[27] Xu F., Zheng Z., Yao M., Zhu F., Shen T., Li J., Zhu C., Yang T., Shao M., Wan Z., Fang C. (2022). A regulatory mechanism of a stepwise osteogenesis-mimicking decellularized extracellular matrix on the osteogenic differentiation of bone marrow-derived mesenchymal stem cells. J. Mater. Chem. B.

[28] Luo B., Wang S., Song X., Chen S., Qi Q., Chen W., Deng X., Ni Y., Chu C., Zhou G., Qin X., Lei D., You Z. (2024). An encapsulation-free and hierarchical porous triboelectric scaffold with dynamic hydrophilicity for efficient cartilage regeneration. Adv. Mater. (Deerf. Beach Fla,).

[29] Chen Y., Lee K., Yang Y., Kawazoe N., Chen G. (2020). PLGA-collagen-ECM hybrid meshes mimicking stepwise osteogenesis and their influence on the osteogenic differentiation of hMSCs. Biofabrication.

[30] Manzano M., Vallet-Regí M. (2025). Mesoporous silica nanoparticles in biomedicine: advances and prospects. Adv. Mater..

[31] Liu Y., Zhao M., Zhang M., Yang B., Qi Y.-K., Fu Q. (2025). Mesoporous silica nanoparticle-based nanomedicine: preparation, functional modification, and theranostic applications. Mater. Today Bio.

[32] Mora-Raimundo P., Lozano D., Benito M., Mulero F., Manzano M., Vallet-Regí M. (2021). Osteoporosis remission and new bone formation with mesoporous silica nanoparticles. Adv. Sci..

[33] Deng R., Zhao R., Zhang Z., Chen Y., Yang M., Lin Y., Ye J., Li N., Qin H., Yan X., Shi J., Yuan F., Song S., Xu Z., Song Y., Fu J., Xu B., Nie G., Yu J.-K. (2024). Chondrocyte membrane–coated nanoparticles promote drug retention and halt cartilage damage in rat and canine osteoarthritis. Sci. Transl. Med..

[34] Marie P.J., Haÿ E., Saidak Z. (2014). Integrin and cadherin signaling in bone: role and potential therapeutic targets. Trends Endocrinol. Metabol..

[35] Zhang Z., Sha B., Zhao L., Zhang H., Feng J., Zhang C., Sun L., Luo M., Gao B., Guo H., Wang Z., Xu F., Lu T.J., Genin G.M., Lin M. (2022). Programmable integrin and N-cadherin adhesive interactions modulate mechanosensing of mesenchymal stem cells by cofilin phosphorylation. Nat. Commun..

[36] Li T., Zhao J., Yuan J., Ding R., Yang G., Cao J., Zhao X., Liu J., Liu Y., Xu P., Deng J., Miao X., Cheng X. (2025). Harnessing engineered exosomes as METTL3 carriers: enhancing osteogenesis and suppressing lipogenesis in bone marrow mesenchymal stem cells for postmenopausal osteoporosis treatment. Mater. Today Bio.

[37] Li H., Gong Y., Wang Y., Sang W., Wang C., Zhang Y., Zhang H., Liu P., Liu M., Sun H. (2025). β-sitosterol modulates osteogenic and adipogenic balance in BMSCs to suppress osteoporosis via regulating mTOR-IMP1-adipoq axis. Phytomedicine.

[38] Zhao J., Chen A., Wang R., Qiu D., Chen H., Li J., Zhang J., Wang T., Wang Y., Lin Y., Zhou J., Du Y., Yuan H., Zhang Y., Miao D., Wang Y., Jin J. (2024). Bmi-1 epigenetically orchestrates osteogenic and adipogenic differentiation of bone marrow mesenchymal stem cells to delay bone aging. Adv. Sci..

[39] Pötter E., Bergwitz C., Brabant G. (1999). The cadherin-catenin system: implications for growth and differentiation of endocrine tissues. Endocr. Rev..

[40] Zhu M., Zhang K., Feng L., Lin S., Pan Q., Bian L., Li G. (2021). Surface decoration of development-inspired synthetic N-cadherin motif via Ac-BP promotes osseointegration of metal implants. Bioact. Mater..

[41] Chen O., Zhou Y., Xu Z., Liu X., Zhang D., Bai M. (2025). Engineered biomembrane-camouflaged nanoparticles: promising strategies to treat inflammatory skeletal diseases. J. Contr. Release.

[42] Su X., Su M., Guo E., Zhou Y., Yang X., Li S., Ye Y. (2025). Tissue-resident macrophage membrane-coated nanomedicine for targeted tumor therapy. ACS Nano.

[43] Park N., Kim K.S., Lee S., Choi J.H., Na K. (2025). Enhanced stem cell-mediated therapeutic immune modulation with zinc oxide nanoparticles in liver regenerative therapy. Biomaterials.

[44] Zhang F., Tian Y., Fan Z., Liu D., Dai T., Guo Q., Wang Z., You S., Yue G., Xia R., Du J., Xu Y. (2025). In situ engineering mesenchymal stem cells for osteogenic differentiation to reverse inflammaging and restore periodontal bone homeostasis. Adv. Funct. Mater..

