# Supplementary material for: Stage-specific biomimetic nanoparticles reprogram osteoblast-adipocyte equilibrium for targeted osteoporosis therapy
Source: Bioact Mater. 2026 May 13;64:455–70. doi: 10.1016/j.bioactmat.2026.05.004 (PMC13196569; doi:10.1016/j.bioactmat.2026.05.004)
Supplement: Multimedia component 1 [file mmc1.docx]

1. **Supplementary Figure:**


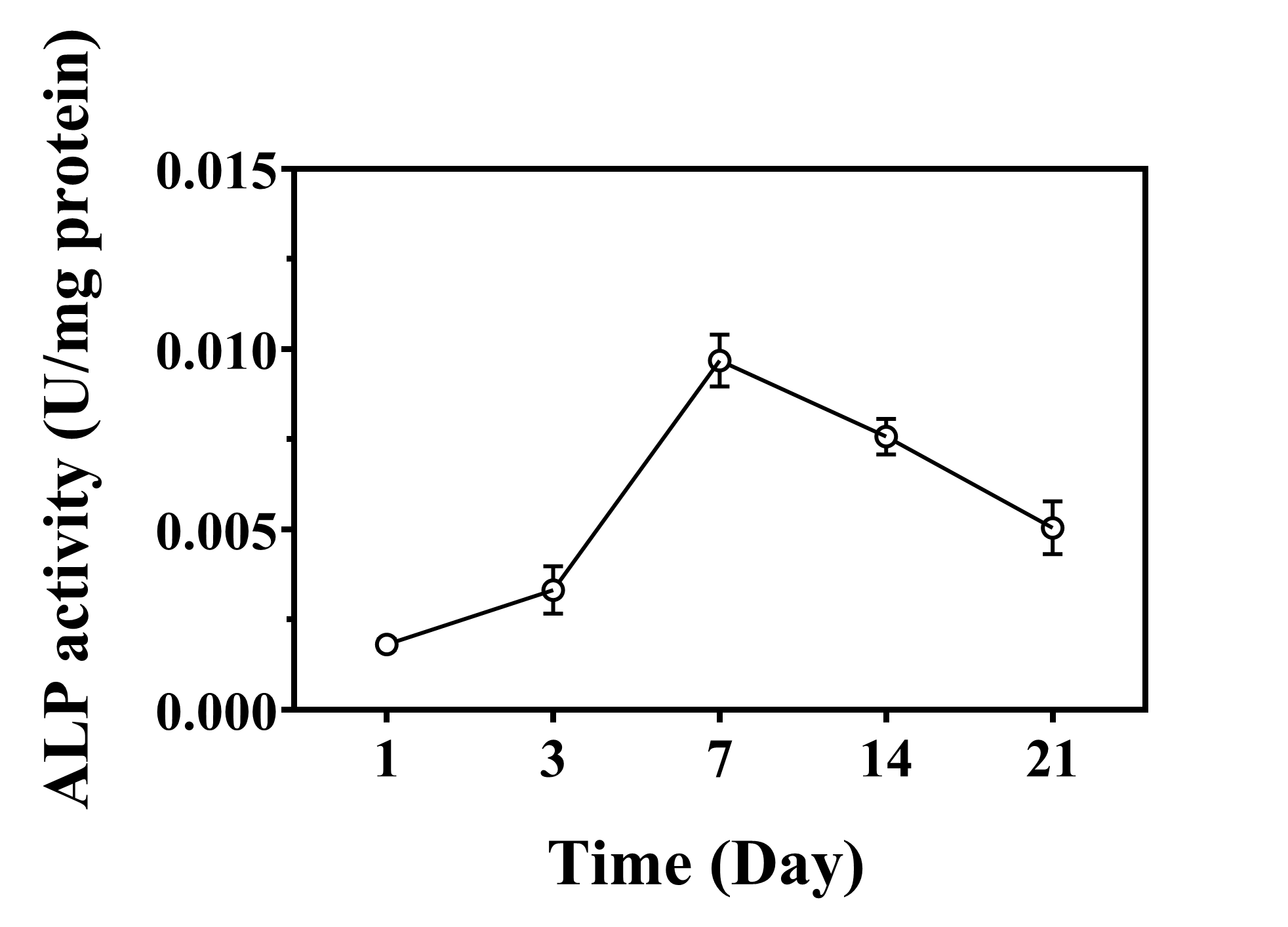


**Fig. S1.** Time-course of ALP activity during osteogenic differentiation of BMSCs from day 0 to 21. Data are presented as mean ± SD (n = 3).


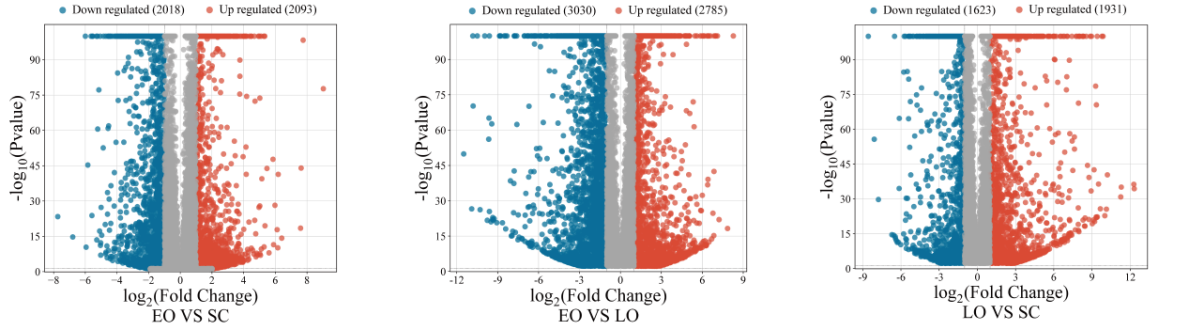


**Fig. S2.** Volcano plots showed differentially expressed genes (DEGs) in each pairwise comparison.


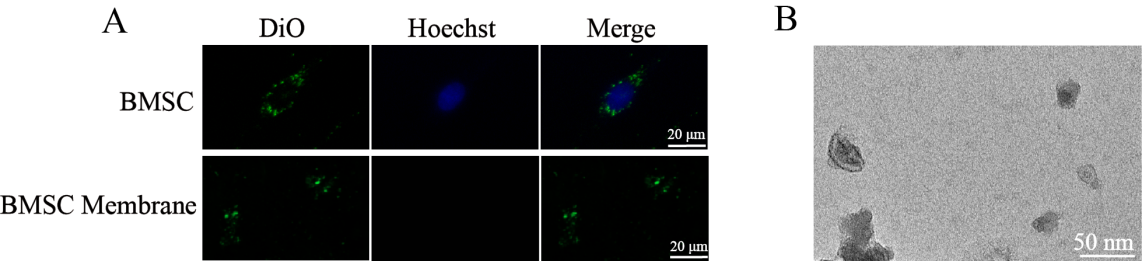


**Fig. S3.** **Validation of membrane purity and structure.** (A) Fluorescence microscopy images showed DiO-labeled membrane (green) and Hoechst-stained nuclei (blue) before extraction, and only DiO signal after isolation, indicating negligible nuclear contamination. (B) TEM image of isolated membrane vesicles showed intact vesicle structures.


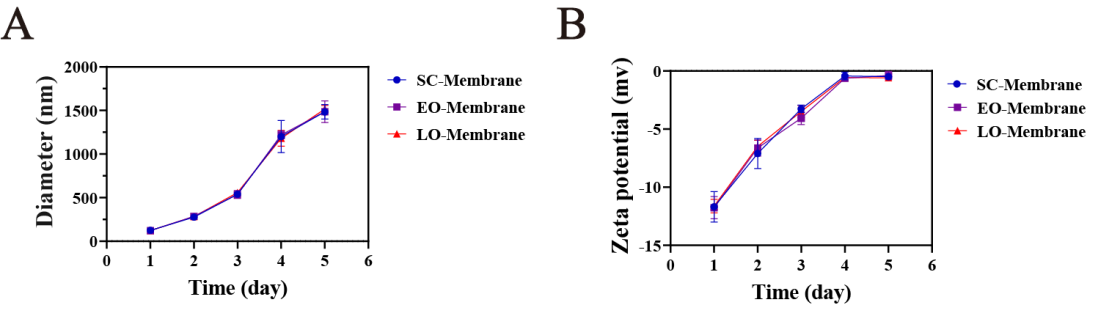


**Fig. S4.** Changes in diameter (A) and zeta potential (B) of different cell membrane over 5 days. Data are presented as mean ± SD (n = 3).


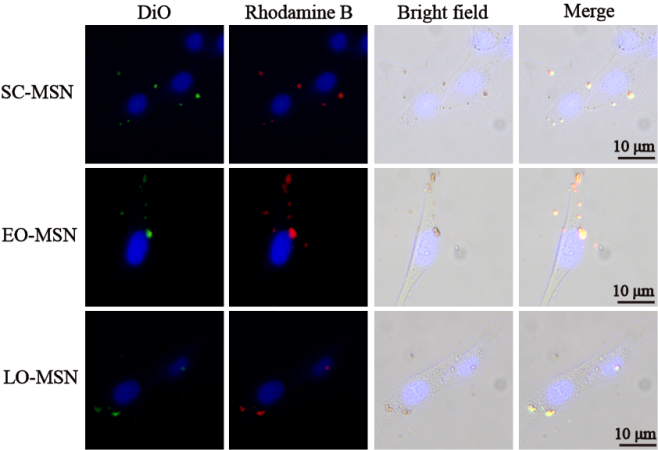


**Fig. S5.** Representative microscopy images of BMSCs treated with different CM-MSNs. MSN cores were encapsulated with Rhodamine B (red). Cell Membranes were labeled with DiO (green) and nuclei with Hoechst (blue).


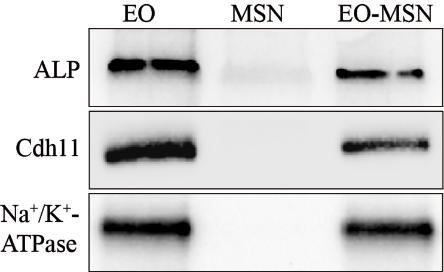


**Fig. S6.** Western blot analysis of membrane protein retention in EO-MSN.


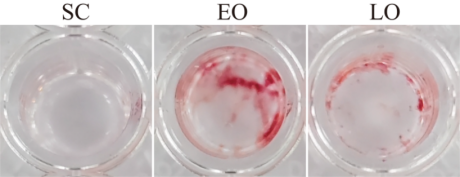


**Fig. S7.** ARS staining images of isolated cell membranes incubated in osteogenic differentiation medium for 21 days.

**
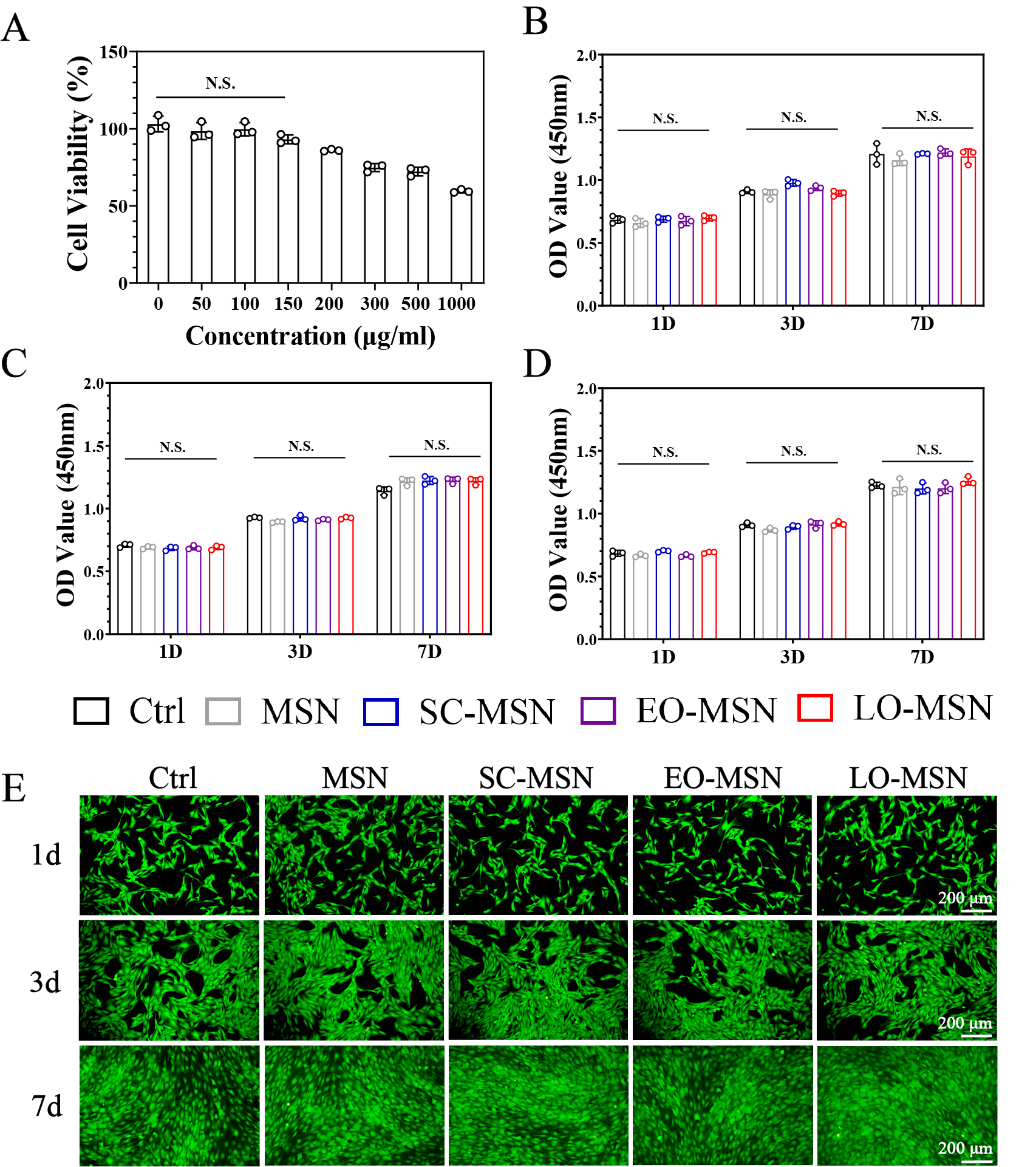
**

**Fig. S8. Biocompatibility of CM-MSN.** (A) CCK-8 analysis of BMSCs co-cultured with different concentrations of MSN after 24 h. CCK8 analysis of BMSCs co-cultured with different CM-MSN at 50 μg/ml (B), 100 μg/ml (C), 150 μg/ml (D) concentration for 1, 3, 7 days. (E) Live cell staining images of BMSCs co-cultured with different CM-MSNs at 150 μg/mL for 1, 3 and 7 days. Data are presented as mean ± SD. N.S., no significant difference.

**
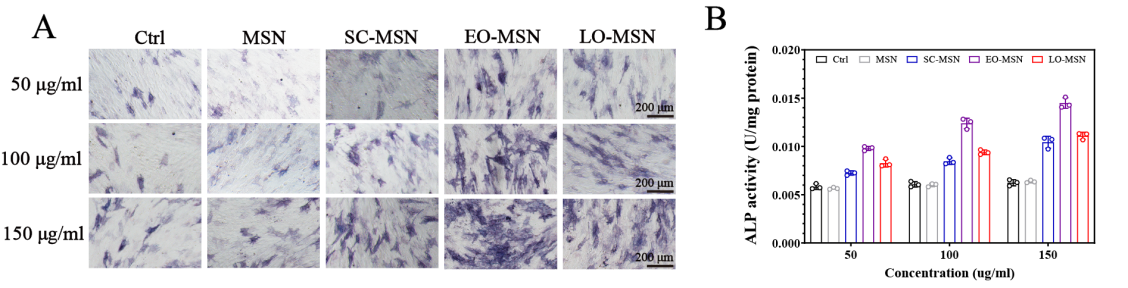
**

**Fig. S9. Concentration-dependent effects of CM-MSNs on BMSC osteogenesis.** (A) Representative ALP staining images of BMSCs treated with different CM-MSNs at concentrations of 50, 100, and 150 μg/mL. (B) Quantitative analysis of ALP activity under different conditions. Data are presented as mean ± SD (n = 3).


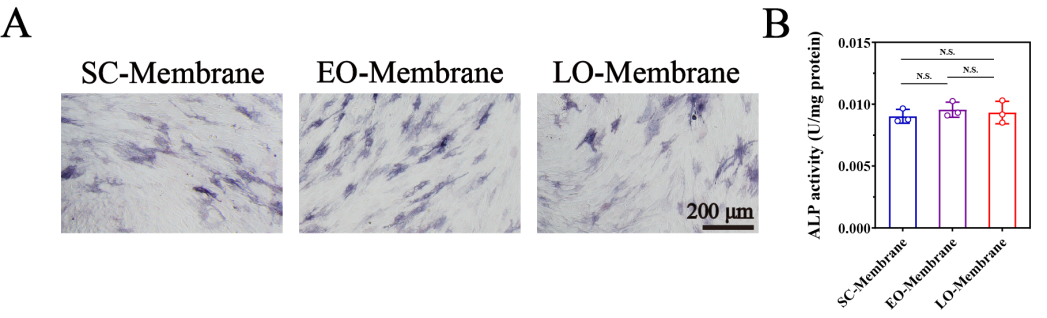


**Fig. S10.** (A) ALP staining images of different groups. (B) Quantitative analysis of ALP activity in different groups. Data are presented as mean ± SD (n = 3). N.S., no significant difference.


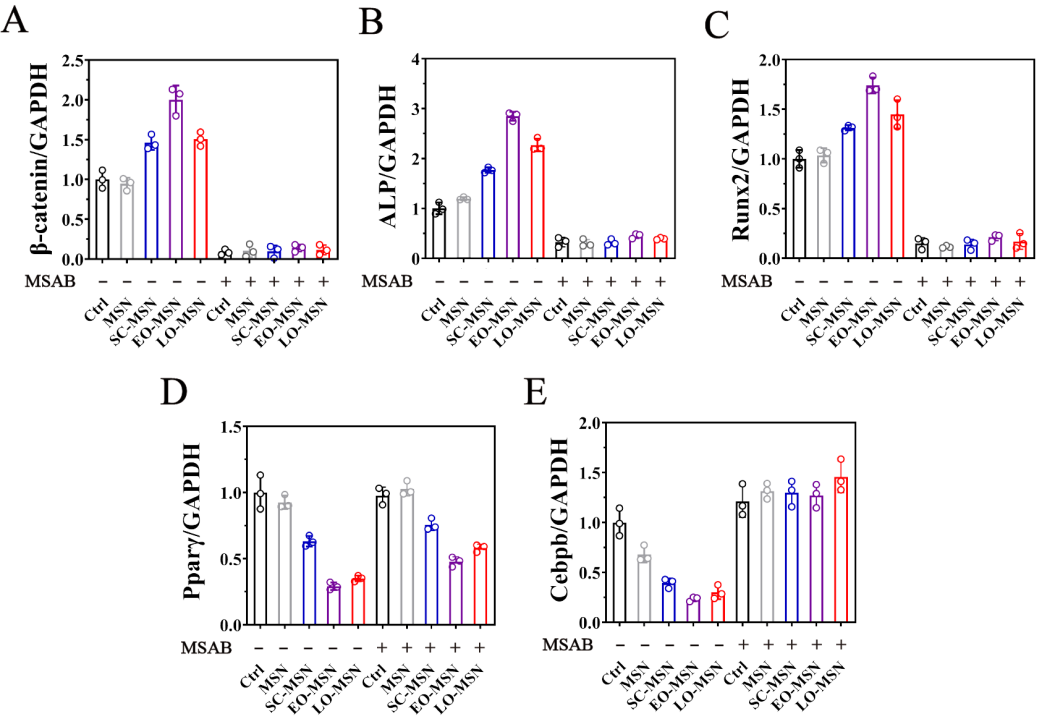


**Fig. S11.** **Quantitative analysis of Western blot with or without MSAB treatment.** (A) β-catenin (B) ALP (C) Runx2 (D) Pparγ (E) Cebpb. Data are presented as mean ± SD (n = 3).


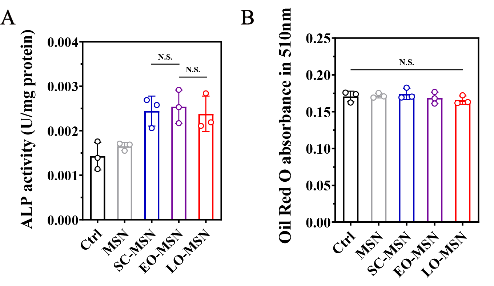


**Fig. S12. Quantitative analysis of osteogenic and adipogenic staining following MSAB treatment.** (A) Quantitative analysis of ALP activity in different groups treated with MSAB. (B) Quantitative analysis of Oil Red O staining in different groups treated with MSAB. Data are presented as mean ± SD (n = 3). N.S., no significant difference.


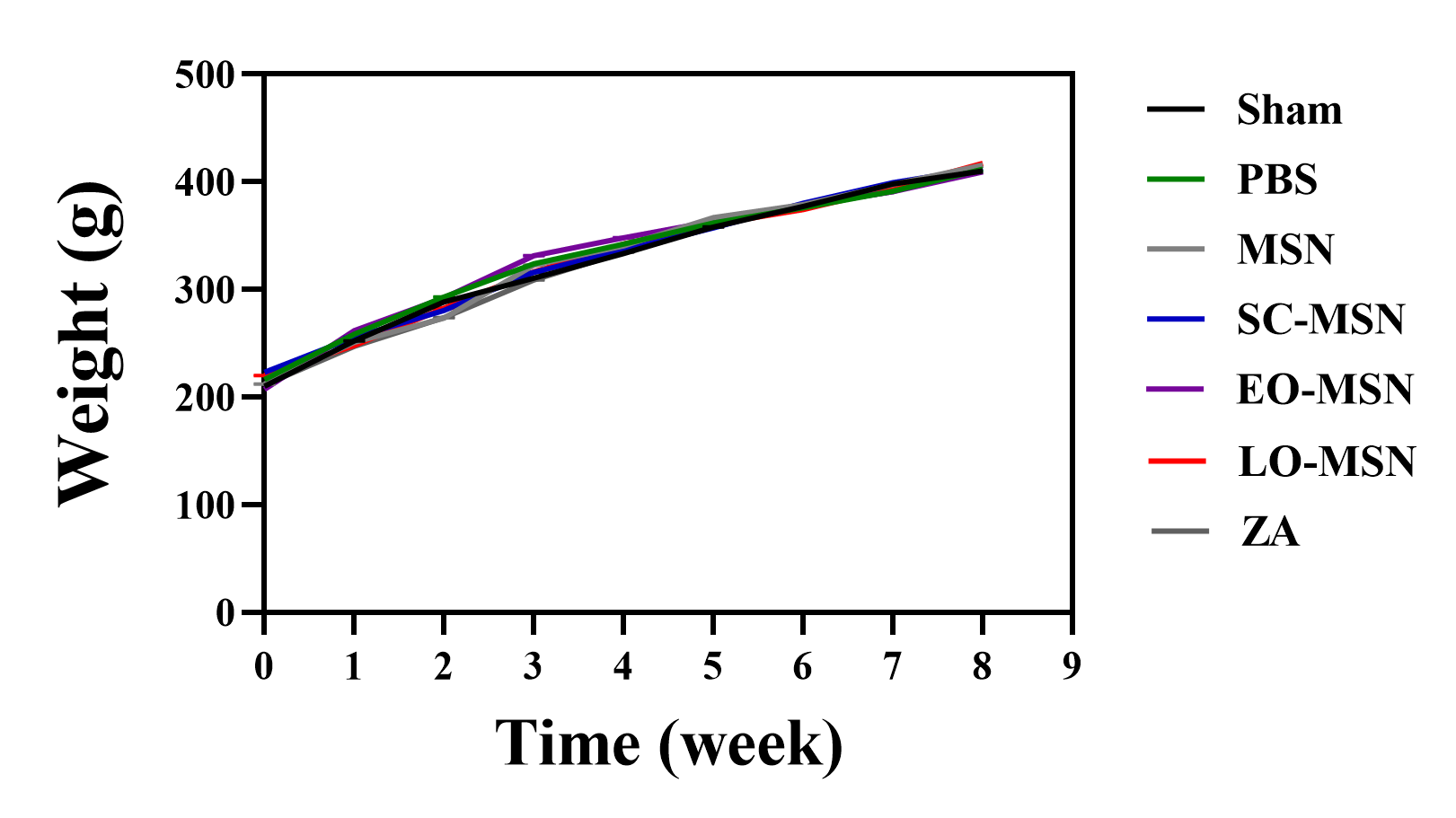


**Fig. S13.** Bodyweight of rats after ovariectomy and different treatment. Data are presented as mean ± SD (n = 6).


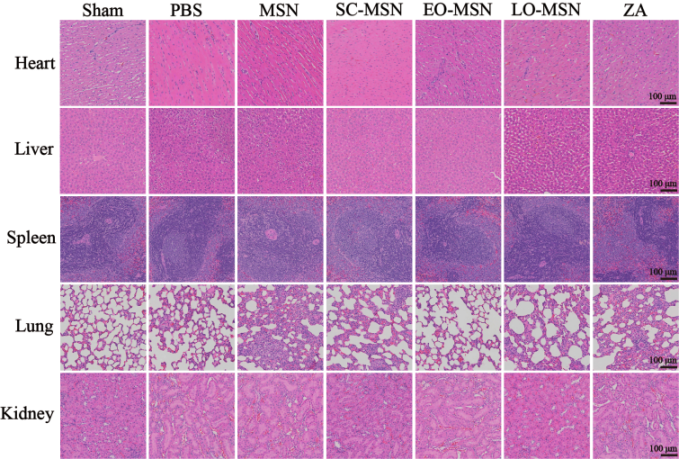


**Fig. S14.** H&E staining of the heart, liver, spleen, lung, and kidney obtained from rats after different treatments.


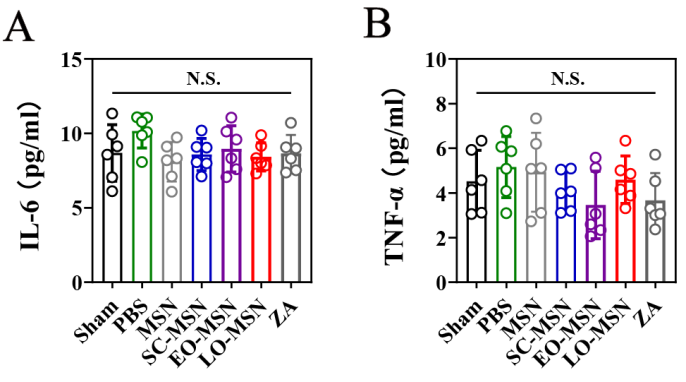


**Fig. S15.** **Serum inflammatory cytokine levels after different treatment.** ELISA quantitative analysis of IL-6 (A) and TNF-α (B) in serum collected from Sham, PBS, MSN, SC-MSN, EO-MSN, LO-MSN, and ZA groups. (n = 6). Data are presented as mean ± SD (n = 6). N.S., no significant difference.


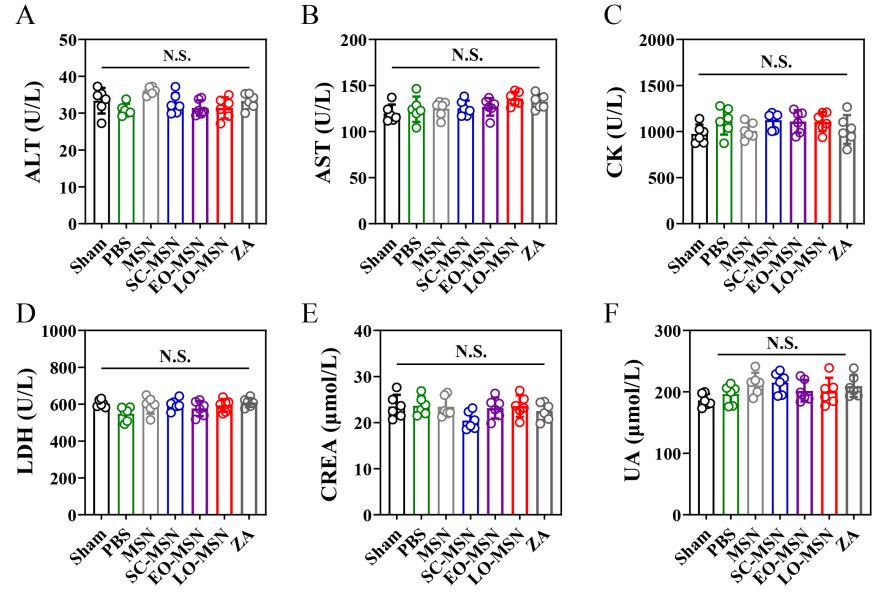


**Fig. S16. The serum biochemical analysis of rats after different treatments.** (A) ALT, alanine aminotransferase; (B) AST, aspartate transaminase; (C) CK, creatine kinase; (D) LDH, lactate dehydrogenase; (E) CREA, creatinine; (F) UA, uric acid. Data are presented as mean ± SD (n = 6). N.S., no significant difference.

1. **Supplementary Table**

**Table S1.** Antibodies used in this study.

| **Antibody** | **Catalog Number** |
| --- | --- |
| Rabbit anti-SMPD3 Polyclonal Antibody | abs133841 |
| Rabbit anti-CDH11 Polyclonal Antibody | abs118321 |
| Rabbit anti-E-Cadherin Recombinant  Monoclonal Antibody | abs172311 |
| ALPL Polyclonal antibody | 11187-1-AP |
| ATP1A1 Polyclonal antibody | 14418-1-AP |
| Beta Catenin Polyclonal antibody | 51067-2-AP |
| GAPDH Polyclonal antibody | 10494-1-AP |
| Collagen Type I Polyclonal antibody | 14695-1-AP |
| Runx2 Polyclonal antibody | 20700-1-AP |
| Osteocalcin Rabbit mAb | A20800 |
| CEBPB Monoclonal antibody | 66649-1-Ig |
| PPAR Gamma Polyclonal antibody | 16643-1-AP |
| LEF1 Polyclonal antibody | 14972-1-AP |
| TCF7L2 Polyclonal antibody | 13838-1-AP |

**Table S2.** Primers used in this work.

| **Name** | **Sequence** |
| --- | --- |
| Alp Forward | AGCAGCATCTTACCAGTTGTGTCTC |
| Alp Reverse | AAGTAGTTCACATCCTGCGGTTCAG |
| Runx2 Forward | TTCAGAACTGGGCCCTTT |
| Runx2 Reverse | CTCAGTGAGGGATGAAATGC |
| IBSP Forward | CAGGACTGCCGAAGGAAGAAA |
| IBSP Reverse | GGCCGGTACTTAAAGACCCC |
| OCN Forward | TCAACAATGGACTTGGAGCC |
| OCN Reverse | TCGAGTCCTGGAGAGTAGCC |
| Cebpb Forward | TGCACCGCAAGTGCTTCTAG |
| Cebpb Reverse | TTTCTGCGCCGTTAGGTTTC |
| Pparg Forward | CGGTTGATTTCTCCAGCATT |
| Pparg Reverse | TCGCACTTTGGTATTCTTGG |
| β-catenin Forward | CCGTTCGCCTTCATTATGGACTAC |
| β-catenin Reverse | GGGCAAAGGGCAAGGTTTCG |
| Lef1 Forward | AGGCGGCGTTGGACAGATC |
| Lef1 Reverse | CTGACAGTGAGGATGGGTAGGC |
| Tcf7l2 Forward | GCTAACGACGAACTGATCTCCTTC |
| Tcf7l2 Reverse | ATTGACCAACGACGACTTGACATC |
| GAPDH Forward | CATCACTGCCACCCAGAAGACTG |
| GAPDH Reverse | ATGCCAGTGAGCTTCCCGTTCAG |
